# Incomplete functional divergence drives MADS-box gene synergy during floral development in tomato

**DOI:** 10.1371/journal.pbio.3003946

**Published:** 2026-08-18

**Authors:** Natalia Gaarslev, Ying Xu, Eléonore Lizé, Irene Julca, Natasha Glover, Sebastian Soyk

**Affiliations:** 1 Department of Plant Molecular Biology, University of Lausanne, Lausanne, Switzerland; 2 Center for Integrative Genomics, University of Lausanne, Lausanne, Switzerland; 3 SIB Swiss Institute of Bioinformatics, Lausanne, Switzerland; 4 Department of Computational Biology, University of Lausanne, Lausanne, Switzerland; University of Cambridge, UNITED KINGDOM OF GREAT BRITAIN AND NORTHERN IRELAND

## Abstract

Genetic synergy arises from interactions between functionally related genes that control complex traits in plants and animals. Synergy occurs when the combined effect of multiple genes exceeds the additive contribution from each individual gene. However, genetic mechanisms that drive and maintain synergy remain underexplored. Here, we investigated synergistic interactions among *SEPALLATA* (*SEP*) MADS-box genes during floral development in tomato. We discovered that *SEP* gene synergy emerges from duplicated genes that partitioned functions to regulate inflorescence architecture and floral organ identity. Moreover, synergistic interactions are reflected in non-additive expression changes that coordinate successive developmental stages. Finally, we demonstrate that *SEP* gene synergy occurred due to residual redundancy on the dose-sensitive *FALSIFLORA/ANANTHA* (*FA/AN*) module guiding floral identity. These results indicate that *SEP* gene synergy emerged as a consequence of incomplete functional divergence under gene dosage constraints. Our work provides insights into mechanisms through which gene families diverge to produce the substrate for biological innovations during evolution.

## Abstract 2

Genetic synergy among functionally related genes underlies complex trait formation, yet its mechanistic basis remains unclear. This study shows that MADS-box gene synergy arises from incomplete functional divergence and dosage-sensitive redundancy in tomatoes.

## Introduction

Combining gene mutations can lead to synergistic effects that exceed the expected cumulative effects of each mutation alone. As a result, synergistic interactions can catalyze major leaps in trait adaptation by enabling more-than-additive changes during evolution [1]. Genetic synergy is frequently observed between mutations in genes that are part of networks controlling growth and development [2]. A common source of synergy is based on functional redundancy that arises from gene duplication [2], which produces redundant paralogs that, in the absence of selective pressure for retention, can follow different evolutionary fates [3]. One of the paralogs may accumulate beneficial mutations to acquire novel functions and neofunctionalize, while the other retains the ancestral function. Alternatively, both paralogs may accumulate mutations to partition the ancestral function and subfunctionalize. Finally, one of the paralogs may accumulate mutations and become a nonfunctional pseudogene. However, even ancient paralogs that functionally diverged are often still co-expressed and maintain some degree of redundancy [3].

**Fig 1:**
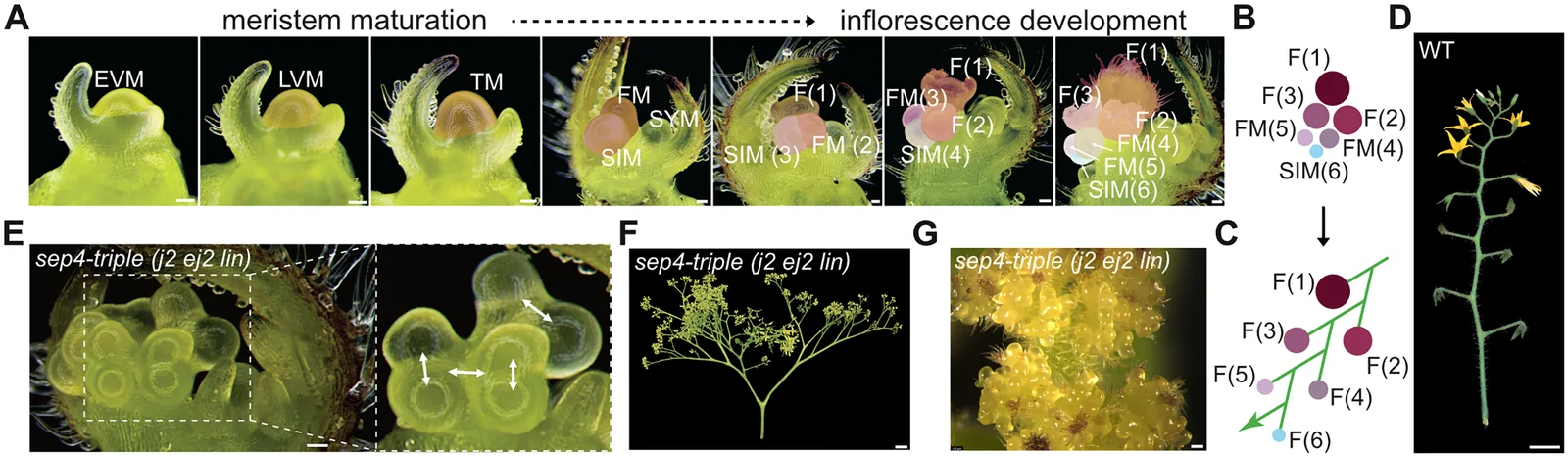
Synergy among tomato *SEP4* paralogs disturbs meristem maturation and leads to inflorescence branching. **(A)** Stereomicroscopy images of a wild-type (WT) shoot apex at subsequent stages of meristem maturation (EVM, early vegetative; LVM, late vegetative; TM, transition; FM, floral; SIM, sympodial inflorescence) and inflorescence development. SYM, sympodial vegetative meristem; F, flower. **(B)** Schematic representation of a developing inflorescence with three flowers, two FMs and one SIM arranged in the characteristic zig-zag pattern. **(C)** Diagram of a mature tomato inflorescence in which six subsequent flowers are arranged in a zig-zag pattern. The arrow represents a new SIM that is formed de novo at the flank of the last flower. **(D)** Representative image of a wild-type tomato inflorescence (cv. Sweet-100). **(E)** Stereomicroscopy images of a *j2 ej2 lin* triple mutant shoot apex after the floral transition with additional inflorescence meristems. The orientation of the release of ectopic meristems is indicated by white arrows in the zoomed image. **(F)** Detached inflorescence of the *j2 ej2 lin* triple mutant showing excessive inflorescence branching and overproliferation of meristematic tissue leading to a cauliflower inflorescence phenotype. **(G)** Stereoscopy image of a *j2 ej2 lin* inflorescence. Scale bars indicate 100 µm (A, E, G) and 1 cm (D, F).

In plants, genetic synergy is frequently observed during the development of flowers and flower-bearing shoots (inflorescences). Flowers develop when shoot apical meristems cease the production of vegetative organs and transition to reproductive development, giving rise to the different organs that constitute a flower. In tomato (*Solanum lycopersicum*) and other sympodial plants, the primary shoot apical meristem progresses from a vegetative meristem (VM) through the transition meristem (TM) and the floral meristem (FM) stage of meristem maturation [4,5], and terminates in the first flower of the multi-flowered inflorescence (Fig 1A). The second flower is produced by a new sympodial inflorescence meristem (SIM) that is initiated at the flank of the first floral meristem to again terminate in a floral meristem and flower. The reiterated process of meristem initiation and termination results in a multi-flowered inflorescence with flowers arranged in zigzag (Fig 1B–1D). The timely control of meristem maturation towards reproductive fate ensures inflorescence development [6]. By contrast, subtle shifts in transcriptional programs that coordinate meristem transitions alter flower production and inflorescence architecture in tomato and other species [7–11]. In tomato mutants, precocious expression programs can result in premature termination of meristems and less complex inflorescences [12,13], while delayed expression programs are associated with the overproliferation of inflorescence meristems that introduce branchpoints in the inflorescence [7,14,15].

Key regulators of meristem maturation have been identified in tomato. Mutations in *FALSIFLORA* (*FA*) [16,17], the ortholog of *Arabidopsis thaliana LEAFY* (*LFY*) [18], the *S/WUSCHEL RELATED HOMEOBOX9* (*S*/*SlWOX9*) [19], and the F-box gene *ANANTHA* (*AN*) [19,20], ortholog of Arabidopsis *UNUSUAL FLORAL ORGANS* (*UFO*) [21] lead to the development of highly branched inflorescence structures that overproliferate meristems and resemble cauliflower curds. Moreover, the three *SEPALLATA* (*SEP*)-clade MADS-box (*MINICHROMOSOME MAINTENANCE1* (*MCM1*), *AGAMOUS* (*AG*), *DEFICIENS* (*DEF*), *SERUM RESPONSE FACTOR* (*SRF*)) transcription factor paralogs *JOINTLESS2* (*J2*), *ENHANCER OF J2* (*EJ2*) and *LONG INFLORESCENCE* (*LIN*) regulate the transition of meristems to floral identity and inflorescence branching patterns [14]. Single *j2*, *ej2*, and *lin* mutants develop mainly unbranched inflorescences, while the *j2 ej2 lin* triple mutants bear cauliflower-like inflorescences that overproduce undifferentiated meristem tissue instead of flowers. The *j2 ej2 lin* triple mutant phenocopies the tomato *an* mutant and shows similarities to the Arabidopsis *apetala1 cauliflower* (*ap1 cal*) double mutants [22–24] (Fig 1E–1G).

*SEP* functions were first reported in the context of floral organ specification [25–27]. Floral organs arise from floral meristems, which typically produce organ primordia that are organized in concentric whorls, with outer vegetative organs, sepals and petals, protecting the inner reproductive organs, the stamens and carpels. Seminal work in Arabidopsis demonstrated that floral organ development is driven by homeotic genes classified into A-, B-, and C-classes [28–30]. In this ABC model, all components, except *APETALA2* (*AP2*), encode MADS-box transcription factors. The A-class genes *AP1* and *AP2* specify sepals while the combination of A-class with the B-class genes *AP3* and *PISTILLATA* (*PI*) specifies petal identity. B-class and the C-class gene *AGAMOUS* (*AG*) determine the identity of stamens, while C-class function determines carpel identity and promotes floral meristem termination. In the early 2000s, the ABC model was expanded to include genes with the E-class function, which is required for proper organ development in all whorls and conferred by the *SEP* MADS-box clade with four members in Arabidopsis [26,27]. At the molecular level, SEP proteins form tetrameric complexes with ABC-class MADS-box proteins in a specific spatiotemporal context to specify distinct floral organ identities [31,32]. MADS-box tetramers consist of two homo- or heterodimers that bind to CArG-box DNA motifs and facilitate DNA looping among distant regulatory elements and target promoters to drive spatiotemporal expression patterns [33].

In Arabidopsis, *SEP* genes act largely redundantly during floral organ development. While *sep3* single mutants show subtle floral organ defects [33], *sep1234* quadruple mutants develop flowers entirely composed of organs with leaf-like identity [26,27]. Studies in other species demonstrated functional divergence among *SEP* paralogs that emerged from *SEP4* duplications [14,34,35]. In addition to *J2*, *EJ2*, *LIN*, tomato encodes a fourth *SEP4* paralog, *RIPENING INHIBITOR* (*RIN*) [14]. *J2* regulates the development of the fruit abscission zone while *EJ2* specifies sepal length and *LIN* regulates the number of flowers per inflorescence [14,36,37]. The fourth *SEP4* paralog, *RIN*, is specifically expressed during fruit maturation and *rin* mutations delay fruit ripening [38] (S1A Fig). Genetic interactions between *J2, EJ2*, and *LIN* lead to synergistic effects on the rate of meristem maturation and inflorescence architecture in higher-order mutants (Fig 1E–1G) [14]. A similar scenario has been described for *SEP4* paralogs in petunia, where the combined loss of *FLORAL BINDING PROTEIN4 (FBP4), FBP9,* and *FBP23* leads to highly branched inflorescences that fail to form flowers [34]. Yet, the genetic and molecular mechanisms that drive synergy among *SEP* paralogs and consequences on gene expression programs underlying inflorescence development remain elusive.

## Results

### Unequal redundancy among *SEP4* paralogs determines tomato inflorescence architecture

**Fig 2:**
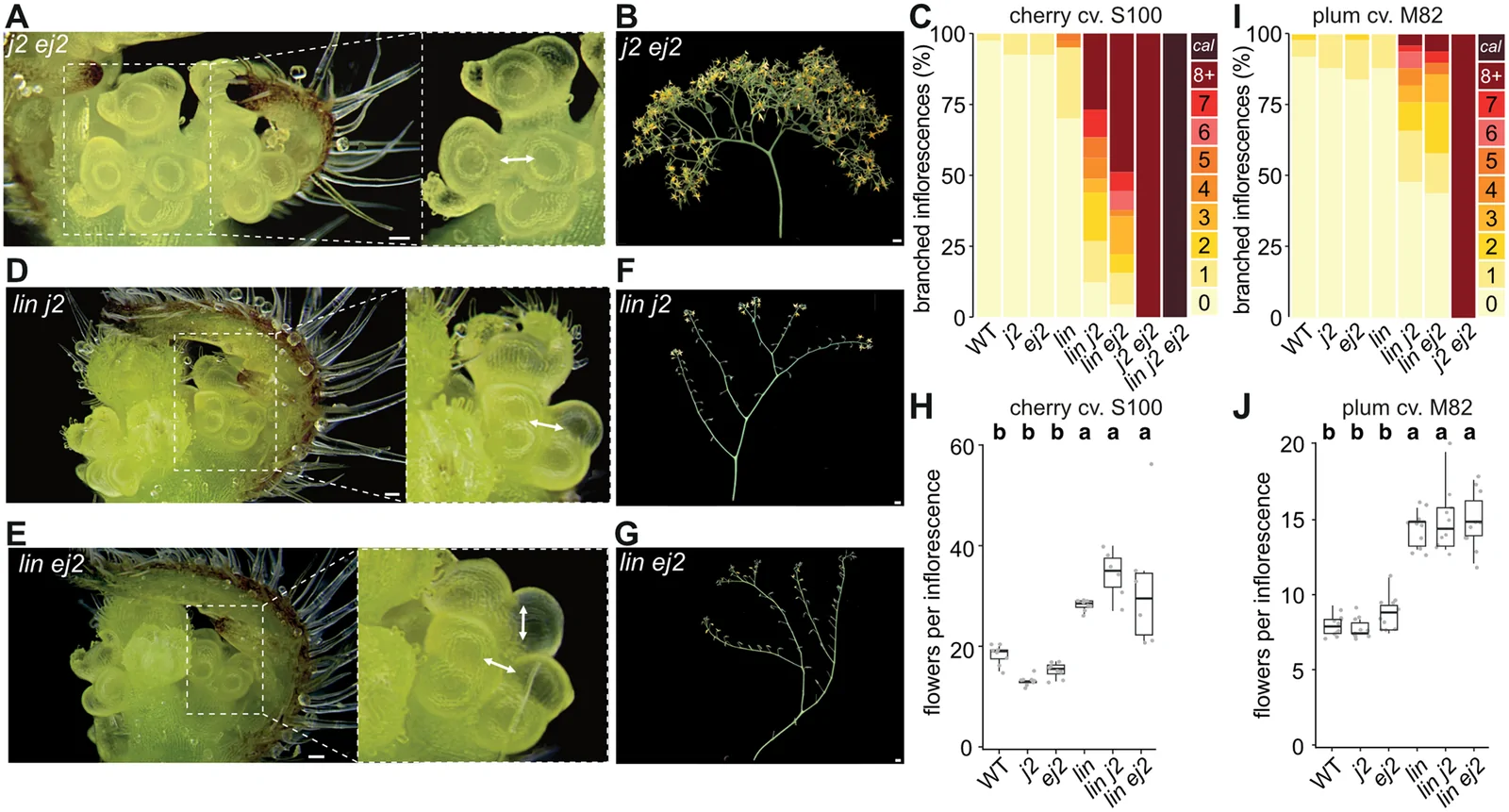
Unequal redundancy among the *SEP4* paralogs *J2*, *EJ2*, and *LIN* suppresses inflorescence branching and flower production in tomato. **(A)** Stereomicroscopy images of a *j2 ej2* double mutant shoot apex after the floral transition with additional ectopic inflorescence meristems on the primary inflorescence. The orientation of de novo release of meristems on the primary inflorescence is indicated by white arrows in the zoomed image. Scale = 100 µm. **(B)** Detached inflorescence of the *j2 ej2* double mutant (cv. S100) displaying strong inflorescence branching. Scale = 1 cm. **(C)** Quantification of inflorescence branching in the S100 cultivar shown as the percentage of branched inflorescences per genotype. cal, cauliflower inflorescence. **(D, E)** Stereoscope images of the shoot apex of *lin j2* (D) and *lin ej2* (E) double mutants. The orientation of the release of ectopic meristems on the inflorescence of the first sympodial shoot is indicated by white arrows in the zoomed image. Scale = 100 µm. **(F, G)** Images of detached inflorescences from the *lin j2* (F) and *lin ej2* (G) double mutants showing weak branching. Scale = 1 cm. **(H)** Quantification of flower number per inflorescence in the S100 cultivar. Note that the *j2 ej2* mutant was excluded due to the unquantifiable (high) flower number. **(I)** Quantification of inflorescence branching in the M82 cultivar as in (C). **(J)** Quantification of flower number per inflorescence in the M82 cultivar as in (H). Letters in (H, J) represent the results from pairwise comparisons of means using one-way ANOVA and post hoc Tukey’s HSD test with 95% confidence level. The data underlying this figure can be found in S1 Data.

Combining mutations in the tomato *SEP4* paralogs *J2*, *EJ2* and *LIN* results in synergistic increases in inflorescence branching and transforms *j2 ej2 lin* triple mutant inflorescences into highly branched, cauliflower-like structures [14] (Fig 1E–1G). To further dissect this example of genetic synergy into the individual contributions of *J2, EJ2,* and *LIN*, we analyzed single and double loss-of-function mutants in a cherry tomato cultivar (cv. Sweet-100) for changes in meristem development and inflorescence branching (S1B Fig). Confirming previous findings, the *j2*, *ej2*, and *lin* single mutants were not markedly affected in inflorescence branching but exhibited specific single mutant phenotypes (S1C–S1F Fig). Fruit abscission was lost in *j2*, sepals were elongated in *ej2*, and inflorescences developed additional flowers in *lin* [14]. In addition, *j2 ej2* double mutants produced ectopic inflorescence meristems, which resulted in strongly branched inflorescences on primary and sympodial shoots bearing flowers with elongated sepals and missing abscission zones [14] (Fig 2A–2C). In comparison, *lin j2* and *lin ej2* double mutants initiated fewer ectopic inflorescence meristems, which led to weakly branched inflorescences at high penetrance on sympodial shoots and rarely on primary shoots (Fig 2C–2G). The *lin j2* and *lin ej2* double mutants also developed more flowers per inflorescence compared to WT, but were not markedly enhanced compared to *lin* single mutants (Fig 2H). In addition, *lin j2* lacked fruit abscission zones while *lin ej2* developed elongated sepals, reminiscent of the *j2* and *ej2* single mutants (S1G Fig). These observations indicate synergistic interactions between *LIN* and both *J2* and *EJ2* during inflorescence development; however, the effect was weaker when compared with synergy between *J2* and *EJ2* [14]. To account for genotype dependencies, we also revisited a previously published mutant collection in a plum tomato (cv. M82) [14], which confirmed weak inflorescence branching and more flowers per inflorescence in *lin j2* and *lin ej2* (Figs 2I, 2J, and S1G). In conclusion, we resolved synergistic interactions in *lin j2* and *lin ej2* double mutants that are weaker compared with interactions in *j2 ej2.* These results indicate unequal redundancy between *SEP4* paralogs with a minor contribution of *LIN* to the suppression of inflorescence branching.

### *SEP4* synergy acts through a limited set of genes after meristems transition to floral fate

**Fig 3:**
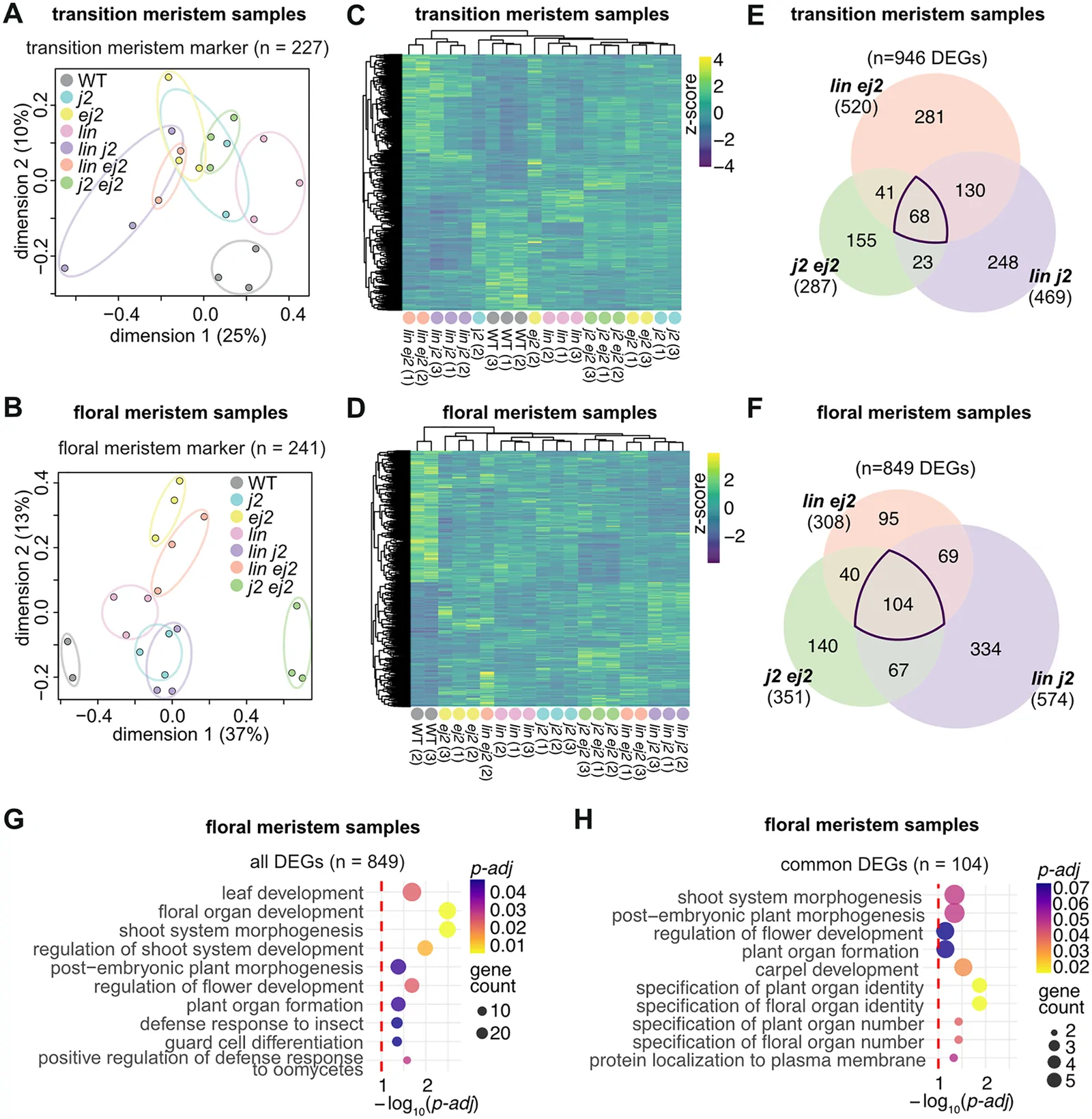
*SEP4* synergy acts on a shared module of genes after the transition stage of meristem maturation. **(A, B)** Multidimensional scaling (MDS) plots of gene expression of 227 transition marker genes in transition meristem samples (A) and 241 floral marker genes in floral meristem samples (B). **(C, D)** Heatmap showing z-score normalised expression of differentially expressed (FC ≥ 1.5, FDR ≤ 0.01) genes in transition (*n* = 946) (C) and floral (*n* = 849) (D) meristem samples. **(E, F)** Overlap of genes differentially expressed in transition (E) and floral (F) meristem samples in *j2 ej2*, *lin j2*, and *lin ej2* double mutants compared with the WT. The total number of DEGs per genotype is indicated in parenthesis. **(G, H)** The 10 most enriched gene ontology (GO) categories for all 849 (G) and common 104 (H) genes differentially expressed in *j2 ej2*, *lin j2*, and *lin ej2* floral meristem samples. Dot size is proportionate to the count of genes per term. *P* values obtained by Benjamini–Hochberg (BH) method in clusterProfiler. The data underlying this figure can be found in S1 Data.

Inflorescence architecture is determined by the rate at which meristems mature from vegetative to reproductive identity [7,15]. To gain molecular insights into how *SEP4* synergy affects the rate of meristem maturation, we sequenced mRNA from micro-dissected meristems of the single and double *sep4* mutants. We sampled meristems at the transition stage of meristem maturation (hereafter transition meristem samples), as well as a combined floral meristem/sympodial inflorescence meristem stage (hereafter floral meristem samples), during which *J2*, *EJ2*, and *LIN* are highly expressed (Figs 1A and S1A). We excluded the *j2 ej2 lin* triple mutant from this experiment due to the difficulty of obtaining staged meristem samples from a segregating triple mutant population. Multidimensional scaling (MDS) analyses of transcriptomes revealed that floral meristem samples clustered by the extent of inflorescence branching (Figs 3A, 3B, and S2) (see Methods). In contrast, transition meristem samples lacked clear clustering patterns, suggesting that floral meristem samples better captured relevant transcriptional changes associated with inflorescence branching. A differential expression analysis contrasting mutant genotypes with the WT yielded a total of 946 and 849 differentially expressed genes (DEGs; |log_2_FC| > 0.58, FDR < 0.01) in the transition and floral meristem samples, respectively (Fig 3C and 3D; S1–S6 Tables). A rather small fraction of DEGs was shared between the three double mutants in the transition samples (68 DEGs, 7.2%) (Fig 3E and S7 Table), but this fraction increased in floral samples (104 DEGs, 12.3%) (Fig 3F and S8 Table). Furthermore, while gene ontology (GO) enrichment analysis of the 946 DEGs from the transition meristem samples did not yield any significantly enriched terms, the 849 DEGs in floral meristem samples showed significant enrichment for GO categories related to morphogenesis, flower development, and floral organ identity (Fig 3G and 3H). We concluded that *SEP4* paralogs retained redundant functions to regulate a defined genetic module during inflorescence and floral meristem maturation stages, at which *SEP4* genes are highly expressed (S1A Fig). Combined loss of *SEP4* activity changes this genetic module and disturbs meristem maturation during the acquisition of floral identity.

### *SEP4* synergy is mirrored by a small group of genes with synergistic expression patterns

**Fig 4:**
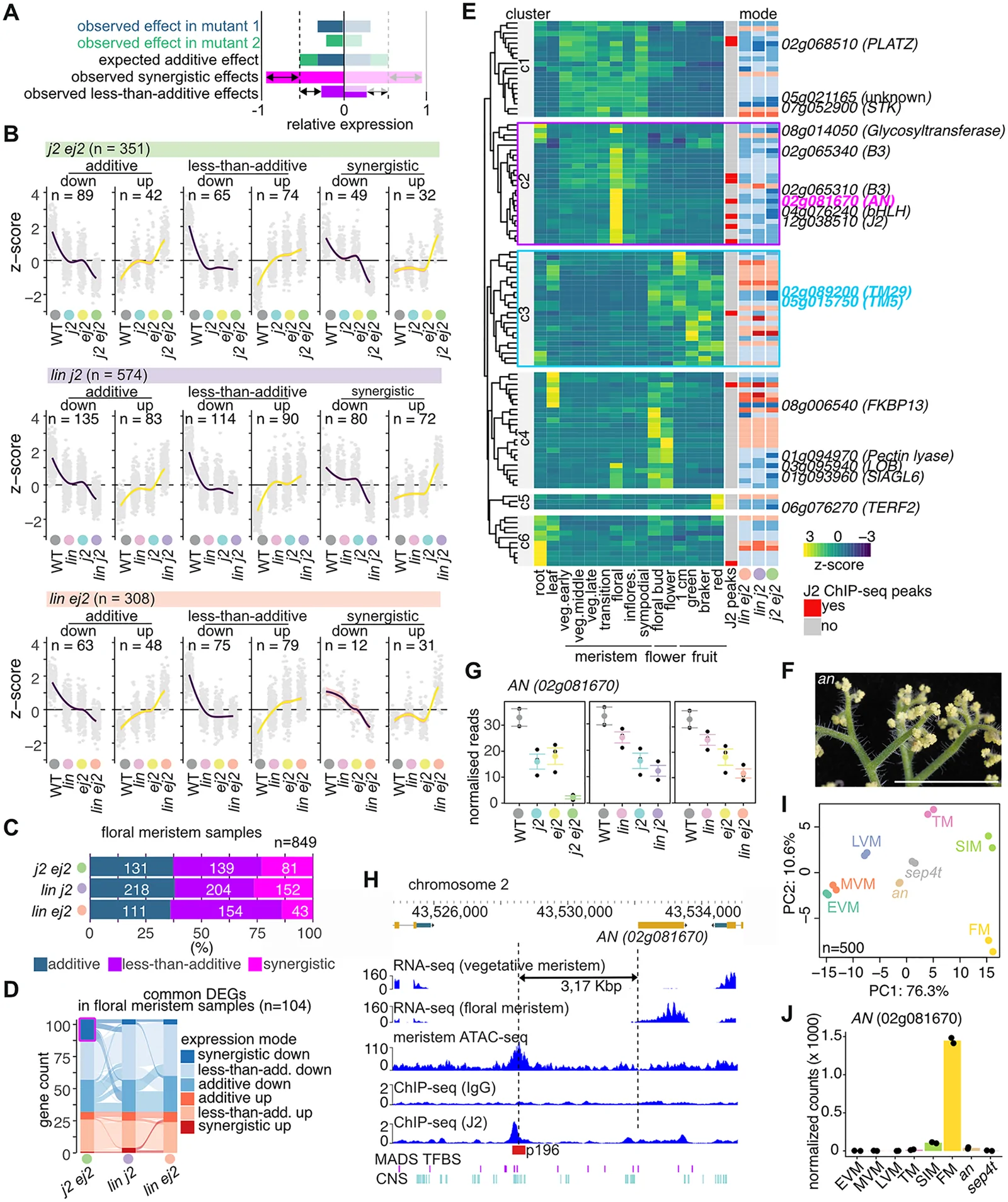
Synergy among *SEP4* paralogs acts on *ANANTHA* to drive the maturation of meristems towards floral fate. **(A)** Schematic representation of additive and non-additive gene expression categories in double mutants when compared with single mutants. **(B)** Normalized expression (z-score) of differentially expressed genes in floral meristems *j2 ej2* (top), *lin j2* (middle), and *lin ej2* (bottom) mutants compared with the WT, classified into additive, less-than-additive, and synergistic (more-than-additive) gene expression categories. Each category is split into genes that are down- and upregulated compared with the WT. Loess-smoothed trendlines are plotted in purple and yellow for down- and upregulated categories, respectively, with 95% confidence intervals shaded in red. n, number of genes per category. **(C)** Distribution of differentially expressed genes in *j2 ej2* (top), *lin j2* (middle), and *lin ej2* (bottom) mutants into additive, less-than-additive, and synergistic (more-than-additive) gene expression categories. **(D)** Overlap of gene expression categories in 104 genes differentially expressed in *j2 ej2*, *lin j2*, and *lin ej2*. **(E)** Normalized gene expression (z-score) for genes in (D) in different tissues and developmental stages. Both expression mode and presence of J2 ChIP-Seq peak are indicated as in (D). Genes synergistically downregulated in *j2 ej2* compared to the single mutants and WT are labeled with gene names. Magenta and blue boxes indicate gene clusters c2 (floral meristem) and c3 (flowers and fruit), respectively. **(F)** Macroscopic image of an *an* mutant inflorescence with overproliferating meristem tissue (cauliflower) instead of flowers. Scale = 1 cm. **(G)** Normalized gene expression of *AN* in floral meristem samples from single and double mutant combinations for *j2 ej2*, *lin j2*, and *lin ej2*. Data are represented as mean ± SE. **(H)** Browser view of the *AN* genomic region. Normalized coverage in counts per million (CPM) is shown for vegetative and floral meristem RNA-seq, meristem ATAC-Seq, and J2 ChIP-Seq (including IgG control). A significant J2 binding peak (P196) is indicated by a red square. Predicted MADS-box transcription factor binding sites (MADS TFBS) and conserved noncoding sequences (CNS) are indicated by magenta and cyan squares, respectively. Genomic positions on chromosome 2 (SL4.0) and the distance between the J2 binding site and the transcriptional start site (TSS) are indicated. **(I)** PCA of the 500 most differential genes in *sep4t* (*j2 ej2 lin*) and *an* inflorescence tissue, and staged WT meristems (EVM, early vegetative; MVM, middle vegetative; LVM, late vegetative; TM, transition; SIM, sympodial inflorescence; FM, floral). **(J)** *AN* expression levels in *an* and *sep4t* inflorescence tissue, and staged WT meristems. The data underlying this figure can be found in S1 Data.

To pinpoint genetic modules that underlie synergistic changes in inflorescence branching, we focused on genes following synergistic expression patterns across floral meristem samples from single and double *sep4* mutants. We adapted an established framework [39] to compare relative expression changes in single and respective double mutants, and isolate genes with non-additive expression patterns that deviate from expected additive effects (Fig 4A; S1–S6 Tables). By including directionality of non-additive expression changes relative to the predicted additive effects, we classified genes into three categories: (1) additive, in which the observed expression change in the double mutant did not significantly differ from the cumulative observed change in the two single mutants; (2) less-than-additive, in which the double mutant change was significantly smaller than the cumulative single mutant change; and (3) synergistic, in which the double mutant change was significantly greater than the cumulative single mutant change. Most genes followed non-additive (less-than-additive or synergistic) expression patterns (Fig 4B and 4C). For example, among the 351 DEGs in the *j2 ej2* floral meristem samples, 220 (63%) followed non-additive expression patterns, including 139 less-than-additive and 81 synergistic genes. Among the non-additive expression changes, we observed more less-than-additive than synergistic changes. DEGs with less-than-additive expression are likely controlled by MADS-box heterocomplexes that contain multiple SEP4 paralogs. In such cases, loss of a single *SEP4* paralog could already cause a reduction in heterocomplex activity that is not further enhanced in the double mutant. On this basis, less-than-additive DEGs are unlikely to explain the synergistic inflorescence phenotypes that are only observed in the double mutants. We concluded that synergistic inflorescence phenotypes are mirrored by widespread non-additive expression changes, but only a small set of genes with synergistic changes drive these complex expression changes.

### *SEP4* synergy acts on the conserved floral meristem identity gene *ANANTHA*

To further investigate synergistic gene expression patterns that are associated with changes in inflorescence branching, we focused on the 104 DEGs that were shared between double mutants in floral meristem samples (Fig 4D and S8 Table). Among those shared DEGs, the *j2 ej2* double mutant yielded the largest number of synergistic DEGs, consistent with the strong, synergistic inflorescence phenotype (Fig 2B). Most synergistic DEGs were downregulated in the *j2 ej2* mutant but downregulated with additive or less-than-additive patterns in the *lin j2* and *lin ej2* combinations, in agreement with the milder inflorescence phenotypes (Fig 2F and 2G). When we investigated tissue-specific expression patterns of 104 shared DEGs using public expression data, we identified a cluster of genes with peak expression in floral meristems [7,40] (Fig 4E). This floral meristem cluster (c2) included *AN*, a known regulator of floral meristem identity and ortholog of Arabidopsis *UFO* [19,41]. Knockout of *AN* leads to cauliflower-like inflorescences reminiscent of the *j2 ej2 lin* triple mutants (Fig 4F) [19,42]. *AN* expression was synergistically downregulated in *j2 ej2* but additively downregulated in *lin j2* and *lin ej2* genotypes, suggesting that *SEP4* dosage-dependent changes in *AN* expression underlie gradual changes in inflorescence branching (Fig 4G). This model is further supported by a J2 binding site upstream of *AN* that we identified in published Chromatin Immunoprecipitation-sequencing (ChIP-seq) data [43]. Interestingly, the J2 binding site resides in an accessible chromatin region (ACR), which contains conserved noncoding sequences (CNS) and *cis*-regulatory elements (CREs) that were shown to impact *AN* transcription [44] (Fig 4H). Given that J2 and other SEP factors bind to a conserved MADS-box target motif (CArG-box) [43,45], we propose *AN* as a direct transcriptional target of SEP4 factors. To further test this hypothesis, we profiled gene expression of *an* and *j2 ej2 lin* triple mutant (*sep4t*) inflorescences by RNA-seq. Principal component analyses (PCA) of mutant transcriptomes with staged WT meristem profiles [7] revealed similar transcriptional states of *an* and *sep4t* meristems (Fig 4I). In addition, *AN* transcripts were barely detectable in *sep4t* meristems (Fig 4J), suggesting that *sep4t* cauliflower inflorescences can be largely attributed to the loss of *AN* expression. In conclusion, reductions in the dosage of *SEP4* paralogs result in quantitative downregulation of *AN*, which explains the synergistic effect on inflorescence branching. However, other genes with peak expression in floral meristems and similar synergistic expression patterns likely act as additional drivers of *SEP4* synergy.

### *SEP4* synergy is relayed to *SEP1/2/3* paralog expression levels

**Fig 5:**
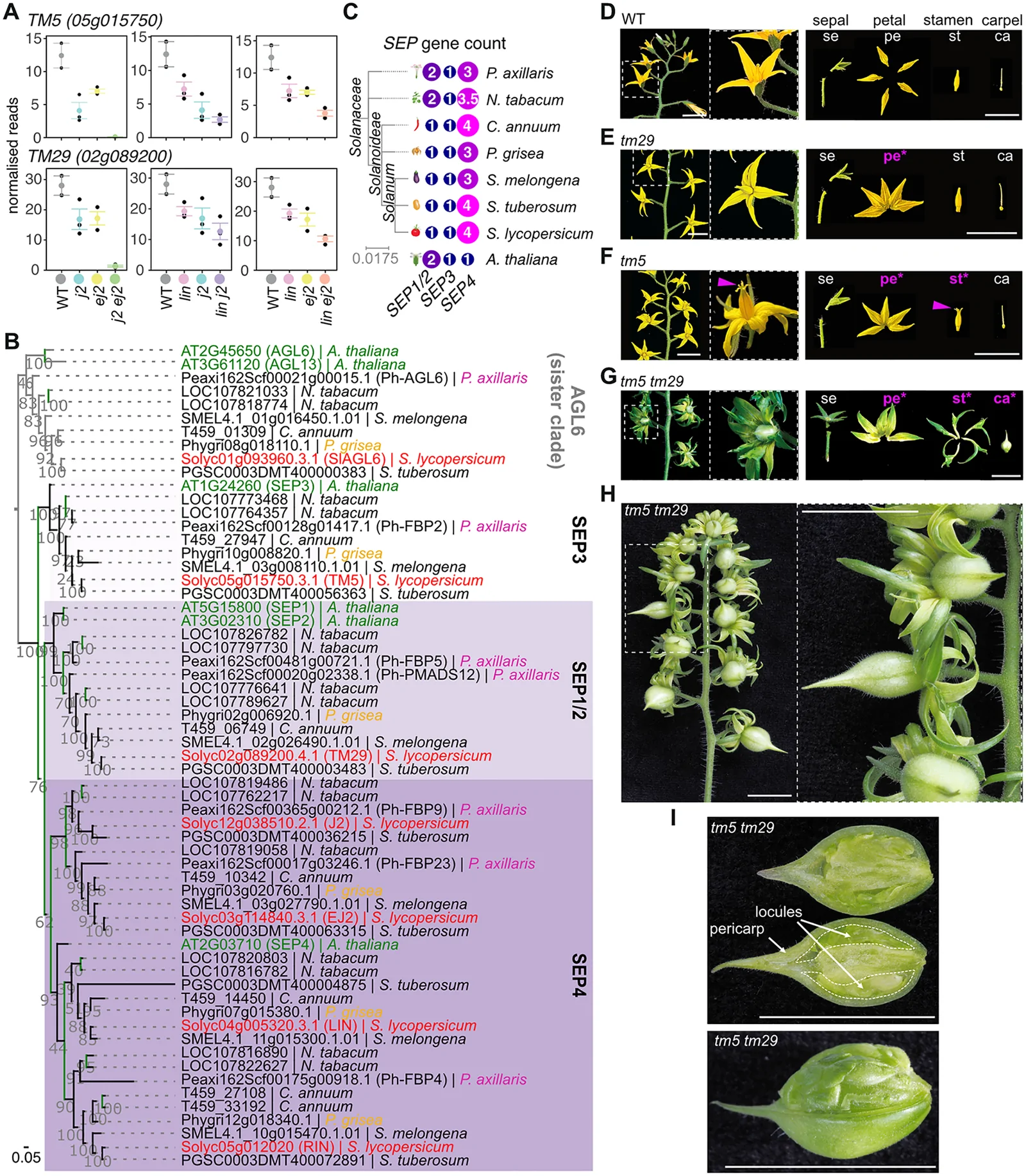
The *SEP1/2* and *SEP3* orthologs *TM29* and *TM5* function redundantly to regulate floral organ identity in tomato. **(A)** Normalized gene expression of *TM5* (top) and *TM29* (bottom) in floral meristem samples from single and double mutant combinations for *j2 ej2*, *lin j2*, and *lin ej2*. Data are represented as mean ± SE. **(B)** Maximum-likelihood tree of SEP proteins in Arabidopsis and Solanaceae. The AGL6 sister clade was used as outgroup. SEP1/2, SEP3, SEP4 subclades are highlighted in different shades of purple. Tomato, physalis, petunia and Arabidopsis genes are labeled in red, yellow, magenta, and green font, respectively. Numbers at nodes represent ultrafast bootstrap support values from 1,000 replicates. Branch lengths are proportional to the number of amino acid substitutions, branches in green show duplication events, and the scale bar indicates the average number of substitutions per site. **(C)** *SEP* gene counts across distinct subclades in Arabidopsis and Solanaceae species shown in the phylogenetic tree in (B). Note that gene content of tetraploid *N. tabacum* is halved to allow comparisons with the other (diploid) genomes. **(D)** Images of a WT inflorescence with a close-up of a single flower (left) and a dissected flower (right), showing the pedicel with calyx (sepals), petals, stamens, and carpels (right). Note the presence of abscission zones on the pedicel of the flower. **(E–G)** Images of the *tm29* (E), *tm5* (F), and *tm5 tm29* (G) mutants as in (D). Note petal fusions in (E–G), bending anthers in (F), and changes in both petal, stamen, and carpel identity in (G) highlighted in magenta font and asterisks. **(H)** Macroscopic image of a *tm5 tm29* double mutant inflorescence with close-up on flowers that produce pseudofruits. **(I)** Macroscopic images of *tm5 tm29* pseudofruits sectioned along the longitudinal axis (top) and with partially removed pericarp (bottom). The pericarp and locules are labeled. Note that locules of pseudofruits are filled with leaf-like organs. Scale bars in (D–I) indicate 1 cm. The data underlying this figure can be found in S1 Data.

In addition to the floral meristem cluster (c2), we identified a cluster (c3) of genes that were primarily expressed in samples derived from developing flowers and fruits, and that included two additional *SEP* paralogs, *TOMATO MADS-BOX5* (*TM5*) and *TM29* (Fig 4E). Previous studies had demonstrated by RNA in-situ hybridization that *TM5* and *TM29* are expressed in floral meristems at the onset of organogenesis and that individual knock-down by antisense technology transforms carpels, the female reproductive floral organs that give rise to the tomato fruit, into ectopic inflorescences [46,47]. *TM5* and *TM29* are lowly expressed during the floral meristem stage of meristem maturation (Fig 4E) and differentially expressed in floral meristem samples across all tomato *sep4* double mutants (Fig 5A), which suggested an additional layer of *SEP* paralog redundancy in floral meristems. Therefore, we revisited the phylogenetic relationship of tomato SEP proteins using hierarchical orthologous groups (HOGs) [48] (Figs 5B and S3A; S9 and S10 Tables). As previously reported [14], we detected an expansion of the SEP family in tomato to six members compared with only four in Arabidopsis (Fig 5B and 5C) [11]. A similar expansion had been observed in the SEP family of the related Solanaceae species petunia [34], and we observed more than four SEP proteins in all other Solanaceae species in our dataset (Fig 5C). This family expansion in Solanaceae is driven by duplication events in the SEP4 clade, with two duplication events that resulted in the paralog pairs J2-EJ2 and LIN-RIN in tomato (S3A and S3B Fig). In contrast, the SEP1/2 clade, which comprises SEP1 and SEP2 in Arabidopsis, is reduced to TM29 in tomato, and this contraction to a single SEP1/2 paralog is conserved throughout the Solanoideae subfamily of Solanaceae (Fig 5B and 5C). Paralog copy number is conserved in the SEP3 clade, which is the ancestral SEP subclade when using the AGAMOUS LIKE6 (AGL6) sister clade as an outgroup, and comprises tomato TM5 and the Arabidopsis ortholog SEP3.

### Redundancy between tomato *SEP1/2* and *SEP3* subclades confers the conserved *SEP* function to specify floral organ identity

Since *TM5* and *TM29* were expressed in floral meristems (S4A Fig) and synergistically downregulated in *j2 ej2* floral meristem samples (Fig 5A), we reasoned that *TM5* and *TM29* may play roles during inflorescence development. Thus, we generated *tm5* and *tm29* mutants and isolated loss-of-function alleles that disrupt the conserved MADS-box domain (S4B Fig). Single *tm5* and *tm29* mutants did not show obvious changes in inflorescence architecture (S4C Fig). Moreover, we observed only very subtle defects in floral organ development: petals on *tm5* and *tm29* mutants were partly fused, anthers in *tm5* flowers were bending outwards, and *tm5* fruits produced fewer seeds (Figs 5D–5F and S4D–S4F). We could not confirm the strong floral organ defects of *tm5* and *tm29* single mutants from previous antisense knock-down studies [46,47]. However, the mild flower defects observed in *tm5* hinted towards unequal redundancy between *TM5* and *TM29* with a more prominent role for the *SEP3* ortholog *TM5* during floral organ specification. Indeed, organ defects were dramatically enhanced in *tm5 tm29* double mutants, which developed flowers with petals and stamen transformed to leaf-like organs (Figs 5G and S4G). In addition, *tm5 tm29* flowers gave rise to parthenocarpic (seedless) fruits that contained leaf-like organs instead of seeds (Figs 5H and 5I and S4H–S4I), similar to petunia *sep123* (*fbp2 fbp5 pmads12*) mutants, which develop ovaries filled ovules converted to leaf-like organs. In contrast to tomato *tm5 tm29*, petunia *fbp2 fbp5 pmads12* flowers retain partial stamen identity [49]. This transformation of floral organs into leaf-like organs in all floral whorls in *tm5 tm29* is reminiscent of the *sep123* triple mutant in Arabidopsis, after which the *SEPALLATA* gene family was named [26]. However, in contrast to Arabidopsis, loss of *SEP1/2/3* activity in tomato still permits pericarp development in *tm5 tm29* mutants. We concluded that *TM5* and *TM29* act redundantly to confer the conserved *SEP1/2/3* function in specifying floral organ identity across all four whorls. Unequal redundancy between *TM5/TM29* during floral organ specification indicates that they are an ancient pair of paralogs that diverged due to compensatory drift [3].

### *SEP4* paralogs retained a function in specifying floral organ identity

**Fig 6:**
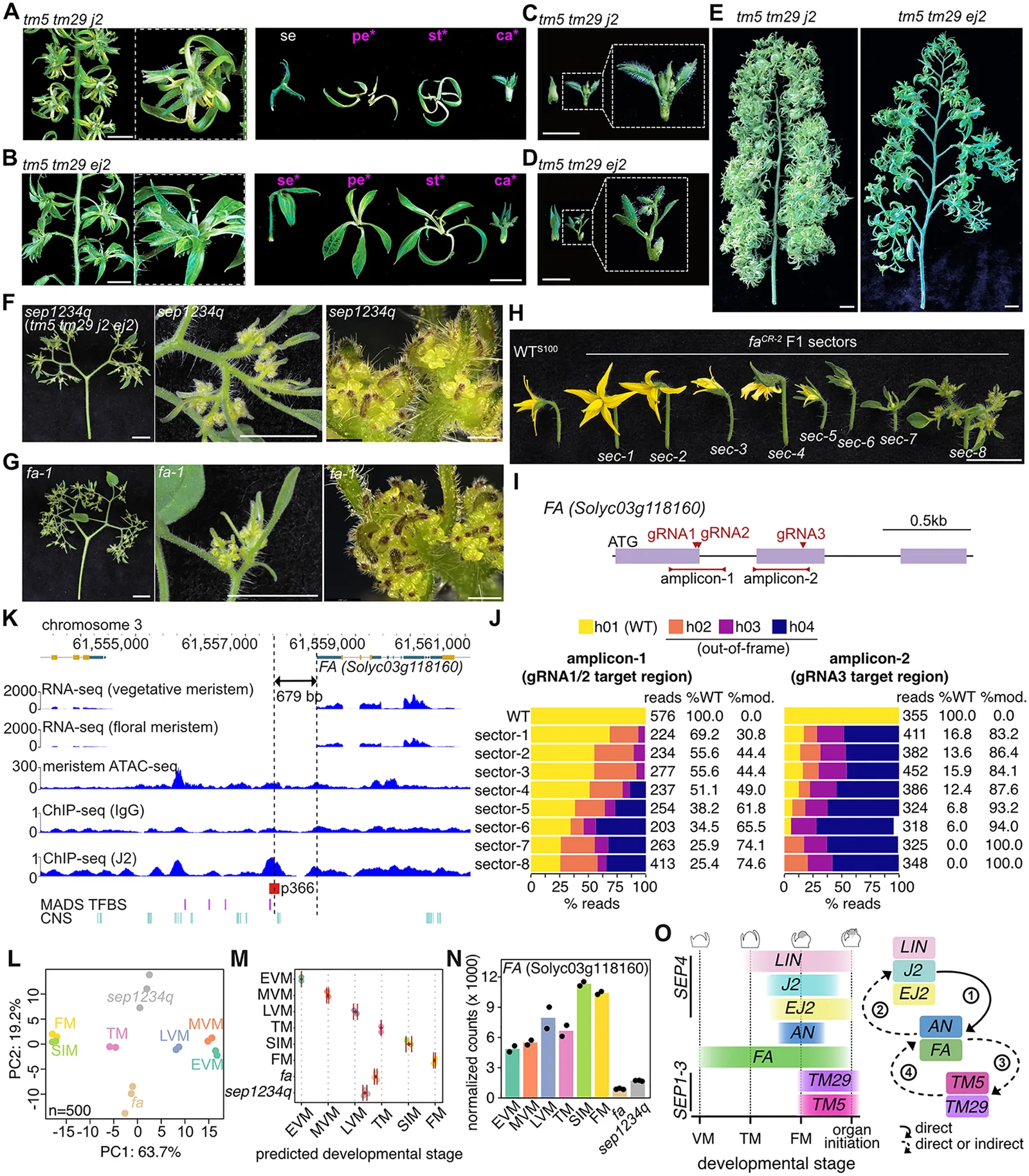
Redundant *SEP1/2/3/4* activity acts on *FALSIFLORA* during the specification of floral organ identities. **(A, B)** Images of *tm5 tm29 j2* (A) and *tm5 tm29 ej2* (B) triple mutants with a close-up of single (left) and dissected (right) flowers, showing the pedicel with calyx (sepals), petals, stamens, and carpels (right). Note the changes in petal, stamen, and carpel identity in (A, B) highlighted in magenta font with asterisks. **(C, D)** Images of dissected *tm5 tm29 j2* (C) and *tm5 tm29 ej2* (D) carpels showing the formation of ectopic inflorescences. **(E)** Images of *tm5 tm29 j2* (left) and *tm5 tm29 ej2* (right) inflorescences showing the reiterated formation of ectopic inflorescences in flowers. **(F, G)** Macroscopic (left and middle) and stereomicroscopic (right) images of a *sep1234q* quadruple (F) and *falsiflora* (G) mutant inflorescences with overproliferation of meristem tissue (cauliflower) and bracts. **(H)** Macroscopic images of detached flowers from different sectors (*sec*) of a *fa*^*CR*^ F1 individual. Note the gradual changes in floral organ identity from *sec-2* to *sec-7*, and the cauliflower inflorescence in *sec-8*. **(I)** Schematic representation of *FA* with guide RNAs (gRNA) target positions indicated by arrowheads. Purple boxes represent exons. The Cas9 cleavage sites for gRNAs are indicated with arrowheads. **(J)** Quantification of *FA* haplotype frequencies in different *fa* sectors and a WT control by amplicon deep sequencing. The total number of reads, percentage of wild-type (%WT) and modified (%mod.) reads are shown. Note that haplotype-1 (h01) represents the WT haplotype at both target regions. **(K)** Browser view of the *FA* genomic region. Normalized coverage (CPM) is shown for vegetative and floral meristem RNA-seq, meristem ATAC-Seq, and J2 ChIP-Seq (including IgG control). A significant J2 binding peak (P366) is indicated in red. Predicted MADS-box binding sites (MADS TFBS) and conserved noncoding sequences (CNS) are indicted in magenta and cyan, respectively. The distance between the J2 binding site and the transcriptional start site (TSS) are indicated. **(L)** PCA of the 500 most differential genes in *fa* and *sep1234q* meristems and staged WT meristems (EVM, early vegetative; MVM, middle vegetative; LVM, late vegetative; TM, transition; SIM, sympodial inflorescence; FM, floral). **(M)** Developmental stage prediction for *fa* and *sep1234q* meristem tissue from transcriptome data using RAPToR. Age estimates for replicate samples are shown. Diamonds and error bars indicate mean values and standard deviation. **(N)** Expression (normalized read counts) of *FA* in staged WT meristems and *fa* and *sep1234q* meristem tissue. **(O)** Proposed regulatory feedback between *SEP* genes and *AN/FA*. Stage-specific expression patterns of *SEP1-4* and *AN*/*FA* (left) support a sequential, positive feedback between *SEP* genes and the *AN*/*FA* module (right): (1) At the transition from vegetative to floral meristem identity, *SEP4* genes redundantly activate *AN*, supported by *sep4*-triple and *an* cauliflower-like inflorescences, low *AN* expression in *sep4* single and combinatorial mutants, and J2 binding upstream of *AN*. (2) *AN/FA* positively feedbacks on *SEP4* to maintain floral meristem identity, supported by downregulation of *SEP4* genes in *an* meristems. (3) *AN/FA* activates *SEP1-3* genes in floral meristems to specify floral organ identity, supported by *SEP1-3* downregulation in *an* and *fa* meristems. (4) *SEP1-4* positively feedbacks on *FA* to maintain floral meristem identity, supported by low *FA* expression in *sep4t* and *sep1234q* meristems. Solid and dashed arrows mark regulation with support for direct (genetic analyses, RNA-seq, ChIP-seq) and indirect regulation (genetic analyses, RNA-seq), respectively. Scale bars indicate 1 cm (A–E, F–G (left), H), 0.5 cm (F–G middle), 500 µm (F–G right). The data underlying this figure can be found in S1 Data.

The fact that neither single nor double *tm5 tm29* mutants developed branched inflorescences contradicted the hypothesis that synergistic reduction of *TM5* and *TM29* expression is responsible for inflorescence branching in *j2 ej2* mutants. We reasoned that the effect of *TM5* and *TM29* on meristem activity only becomes visible when *SEP4* activity is reduced, so we combined *tm5* and *tm29* with *j2* and *ej2* mutations. Indeed, loss of *J2* or *EJ2* activity in *tm5 tm29 j2* and *tm5 tm29 ej2* triple mutants enhanced the vegetative identity of sepals, petals, and stamen (Fig 6A and 6B). Yet, the most dramatic changes were again observed in the fourth floral whorl. While *tm5 tm29* mutants developed parthenocarpic fruits with leaf-like organs, the additional loss of one *SEP4* paralog in *tm5 tm29 j2* and *tm5 tm29 ej2* triple mutants caused the development of ectopic inflorescences in the center of each flower (Figs 6A, 6B, S4J, and S4K). This process is reiterated in each new flower, indicating a reversion from floral to inflorescence meristem identity (Fig 6C–6E). These results show that when *SEP4* function is partially lost in the absence of *SEP1/2/3* (in *tm5 tm29 j2* or *tm5 tm29 ej2*), floral meristems produce three whorls of vegetative organs. Mutant floral meristems fail to terminate in the fourth whorl and instead revert to inflorescence meristem identity, producing an ectopic inflorescence with flowers that reiterate the process. This shows that tomato *SEP4* (*J2* and *EJ2*) can confer the *SEP1/2/3* function in the fourth whorl in the absence of *TM5* and *TM29*, consistent with partial redundancy of *SEP1/2/3/4* during carpel development (S4L Fig).

### *SEP1/2/3* paralogs confer a minor function in driving meristem transitions to floral identity

We further reduced *SEP4* activity in the absence of *SEP1/2* or *SEP3* paralogs by isolating *tm29 j2 ej2* (*sep1/2 sep4*^*partial*^) and *tm5 j2 ej2* (*sep3 sep4*^*partial*^) triple mutants. The triple mutants developed inflorescence branching patterns reminiscent of *j2 ej2* (Figs 2B and S5A). In addition, loss of *TM5* (*SEP3*) or *TM29* (*SEP1/2*) increased the occurrence of vegetative organs (bracts) in the inflorescence and defects in floral organ identity (S5A and S5B Fig). As a result, *tm5 j2 ej2* mutants developed petals instead of stamens, indicating that loss of *J2* and *EJ2* further enhances effects in *tm5* on stamen identity. We also isolated the *tm5 tm29 j2 ej2* quadruple (*sep1234q*) mutant, which developed highly branched inflorescences that failed to differentiate flowers and instead developed modified leaves (bracts) and overproliferative meristematic tissue (Fig 6F). This severe loss of floral identity from the combined mutation of *TM29* (*SEP1/2*), *TM5* (*SEP3*), and *J2/EJ2* (*SEP4*) shows that *SEP1/2/3* paralogs confer floral meristem identity when *SEP4* dosage is reduced. In conclusion, *SEP1/2/3* paralogs retained redundant functions with *SEP4* paralogs in promoting meristem maturation towards floral identity.

### *SEP1/2/3/4* redundancy acts on the conserved floral identity gene *FALSIFLORA*

The inflorescences of *sep1234q* mutants closely resembled those of the historical mutant *falsiflora (fa)* [17], which lacks the activity of the tomato ortholog of Arabidopsis *LFY*, a floral identity gene that is deeply conserved across flowering plants [16,18,50]. Hence, we generated *fa* mutants in cherry tomato (cv. Sweet-100) using CRISPR–Cas and observed the expected *fa* phenotypes in multiple first-generation (T0) plants (Figs 6G and S5D–S5F). Due to high Cas9 editing activity, we recovered only one out of 11 *fa*^*CR*^ T0 individuals that developed a few fertile flowers with pollen for backcrossing to the WT. Cas9-free *fa-null/ +* F1 heterozygotes did not develop obvious floral organ or inflorescence defects (S5G Fig), confirming the recessive nature of the *fa* mutant [17]. However, we identified an F1 individual that retained Cas9 activity and developed distinct sectors with abnormal phenotypes ranging from floral organ defects to *fa-null* inflorescences that overproliferated bracts and meristematic tissue (Figs 6H and S5H). The distinct phenotypic sectors suggested that Cas9 had induced additional *fa* mutations, leading to gradual reductions in *FA* dosage and a spectrum of floral organ and inflorescence defects. We quantified the frequency of *FA* mutations in individual flowers from sector 1 (sec-1) to sec-7 or inflorescence tissue (sec-8) by amplicon deep sequencing (Fig 6I). Remarkably, the frequency of the functional *FA* (WT) haplotype indeed decreased with phenotypic severity, from sectors with mild floral organ defects to sectors with strong organ defects and branched inflorescences, which contained only out-of-frame *fa* mutations (Figs 6J and S5I; S11 Table). These results show that gradual reductions in *FA* gene dosage can lead to both floral organ defects and inflorescence branching. Given the phenotypic similarities between *fa* and *sep1234q* mutants, we reasoned that *SEP1/2/3/4* activity is required for maintaining *FA* expression in reproductive meristems, which guides meristem maturation towards floral fate. This model is supported by a J2 binding site upstream of *FA* with two CarG-box elements (Fig 6K) [43] and a recent report that deletion of this site leads to floral organ defects and mild inflorescence branching [51].

To investigate the functional relationship between *SEP1/2/3/4* and *FA*, we profiled gene expression in *fa* and *sep1234q* inflorescences by RNA-seq and compared mutant transcriptomes with staged WT meristem profiles [7]. Principal component analyses of the 500 most variable genes resolved subsequent stages of meristem maturation on the first component (PC1) (Fig 6L). To estimate the developmental stage of *fa* and *sep1234q* meristem tissue, we applied an established framework [52] and staged mutant transcriptomes on the WT meristem maturation trajectory as a reference (Fig S6 and Methods). This analysis predicted *fa* and *sep1234q* near late vegetative (LVM) and transition (TM) meristem stages, suggesting that *fa* and *sep1234q* meristems revert to a maturation stage that precedes the transition to reproductive development (Fig 6M). In addition, *FA* transcript levels were strongly reduced in *sep1234q* meristems, consistent with a model in which *SEP1/2/3/4* activity is required to maintain *FA* expression (Fig 6N). To further investigate this potential positive maintenance relationship, we examined expression of *FA*, *AN* and *SEP1-4* genes across the *sep4t*, *sep1234q*, *an*, and *fa* RNA-seq datasets (S7A and S7B Fig). In addition to low *AN* transcript levels in *sep4t* (*j2 ej2 lin*) mutants, we found that *J2* and *EJ2* were downregulated in *an*, supporting a positive feedback loop between *SEP4* genes and *AN* (Fig 6O). Further, *TM5* and *TM29* were downregulated in both *an* and *fa*, and *AN* and *FA* were also lowly expressed in *sep4t* and *sep1234q*, indicating a second layer of feedback maintenance between *SEP1-3* and *AN/FA* during floral organ specification. Interestingly, *LIN* showed high expression in *an*, *fa*, and *sep1234q* while *TM29* maintained elevated transcript levels in *sep4t* and *sep1234q*, suggesting potential compensatory mechanisms that warrant further investigation (S7A and S7B Fig). Together, these results indicate that incomplete functional divergence of *SEP* genes ensures redundancy on the dose-sensitive *FA/AN* module, which is conserved across flowering plants to drive meristem maturation during inflorescence and flower development [50].

## Discussion

### *SEP* gene synergy emerged from redundancy on the conserved *FA/AN* module

In this study, we dissected synergistic interactions between closely related *SEP4*-clade MADS-box genes in the model crop tomato. Previous studies showed synergy between the *SEP4*-clade paralogs *J2*, *EJ2*, and *LIN* on the suppression of inflorescence branching [14,53]. Here, we found that *SEP4* synergy on gene expression programs underlying inflorescence branching is more pronounced between the direct paralogs *J2* and *EJ2* than with the distant paralog *LIN*. This pattern is consistent with paralog redundancy generally declining over evolutionary time, likely driven by divergence in coding sequences and gene expression patterns [54]. Transcriptome analyses show that *SEP4* synergy acts through a defined set of genes in floral meristems including *AN*, a conserved floral identity gene and ortholog of Arabidopsis *UFO* [19,50]. By integrating data from previous studies [43,44], we propose a model (Fig 6O) in which SEP4 paralogs directly activate *AN* expression by binding an enhancer element ~3.5 kbp upstream of *AN* when meristems transition to floral identity, a critical window in which inflorescence architecture is determined [7,14,15]. Further, we found that synergy between four *SEP1/2/3/4* paralogs acts through the expression of *FA*, a second deeply conserved floral identity gene and ortholog of Arabidopsis *LFY* [16,22,50]. Remarkably, loss of *SEP1/2/3/4* activity leads to lower *FA* transcript levels, and lowering *FA* gene dosage recapitulates the phenotypic spectrum arising from gradual reduction of *SEP1/2/3/4* dosage. These results indicate that *SEP1/2/3/4* synergy converges to maintain *FA* dosage. We found evidence that SEP1/2/3/4 factors directly regulate *FA* by binding to an upstream *cis*-regulatory element, a model supported by published ChIP-Seq data [43] and CRISPR-Cas deletion of this element, which leads to inflorescence branching and homeotic floral organ conversions [51]. Thus, residual *SEP1/2/3/4* redundancy acts on the *FA/AN* module during subsequent stages of meristem development to drive identity transitions to reproductive development, a function that remained conserved when *SEP* genes functionally diverged. Together, these findings show that *SEP* gene synergy emerged as a relic of functional redundancy.

An open question is which other MADS-box factors interact with SEP proteins to form heterotetrameric complexes that bind to the CArG-box target motifs upstream of *AN* and *FA*. We speculate that SEP proteins interact with AP1/FRUITFULL (FUL)-clade MADS-box proteins that are co-expressed in transition and floral meristems [7,11,55]. This model is supported by published yeast two-hybrid assays showing interactions between the AP1/FUL-like proteins MACROCALYX (MC) and FUL2, and the SEP proteins TM5, J2, EJ2, and RIN [55,56]. A recent study [55] reported that heterotetramers of J2 and either MC or FUL2 bind target DNA in electrophoretic mobility shift assays (EMSAs). We revisited affinity purification-sequencing (DAP-seq) experiments with in vitro-reconstituted heterotetramers from this study but did not find evidence for J2/MC or J2/FUL2 binding at the J2 target sites upstream of *AN* and *FA* identified in in vivo meristem ChIP-seq data [43]. However, genetic evidence supports a role of *MC*, *FUL2*, and the *FUL*-like gene *MBP20* in meristem maturation and floral meristem identity. Combined loss of *FUL2* and *MBP20* function results in a flowering delay and moderately branched inflorescences that resemble those of weak *j2 ej2* mutants [55]. The *mc* mutant also flowers late but produces flowers with elongated sepals similar to *ej2* mutants [55,57]. However, in contrast to *ej2*, *mc* inflorescences revert to vegetative growth after the formation of few flowers and this vegetative reversion is strongly enhanced in *mc ful2 mbp20*, indicating redundant roles of *AP1/FUL* genes in conferring floral identity [55,57]. Together, these findings show that *AP1/FUL*-genes are involved in both guiding meristem transitions and specifying floral organ identity, paralleling the roles of *FA* and *SEP* genes described in this study. This dual function supports a model in which the floral transition and floral organogenesis represent a developmental continuum rather than distinct phases [58].

### Feedback regulation among *SEP* paralogs calibrates their gene dosage

We propose that transcriptional feedback regulation among *SEP* paralogs coordinates subsequent stages of meristem maturation by maintaining a critical dosage of the *FA/AN* module. *SEP4* synergy influences the expression of two closely related *SEP1/2/3* genes, leading to a dose-dependent downregulation of *SEP1/2/3* paralogs in floral meristems when *SEP4* activity is reduced. Whether SEP4 proteins directly activate *SEP1/2/3* paralogs remains to be determined since we could not identify J2 binding sites near *TM5* and *TM29*, suggesting either an indirect relationship or a direct control over longer genomic distances. More likely appears that transcriptional feedback between *SEP4* and *SEP1/2/3* paralogs is bridged by the *FA*/*AN* module to guide meristems during the acquisition of floral identity (Fig 6O). The Arabidopsis orthologs of the transcription factor FA (LFY) and the F-box protein AN (UFO) form a heteromeric transcriptional complex that regulates targets which LFY homomers cannot bind [41]. *UFO* forms a ring-like expression domain in vegetative and inflorescence meristems, which has been proposed to establish a pattern that in combination with local activation of *LFY* facilitates floral meristem initiation and bract suppression [58]. In contrast, tomato *AN* is specifically expressed during the floral stage of meristem maturation while *FA* is expressed throughout meristem maturation including stages that precede the floral transition. FA homomers likely confer the *FA* function in promoting the transition to flowering, consistent with a flowering delay observed in *fa* but not in *an* mutants [16,22]. The activation of *SEP4* paralogs at the floral transition precedes *AN* expression in floral meristems (Fig 6O), and *AN* expression is diminished when *SEP4* activity is reduced. *SEP4* function is thus required for *AN* expression and the formation of putative FA/AN heterocomplexes that drive meristem maturation post-transition towards floral identity. *SEP1/2/3* paralog expression is instead activated in floral meristems during floral organ initiation [11], and a critical *SEP* dosage is essential during these maturation stages to maintain *FA* expression. Progressive loss of *SEP* activity in *tm5 tm29* (*sep123*) and *tm5 tm29 j2 ej2* (*sep1234q*) mutants causes homeotic organ conversions and loss of floral meristem determinacy. Similarly, quantitative reductions in *FA* dosage cause floral organ conversions and loss of floral meristem identity [51], and *FA* expression is substantially reduced in *sep1234q* mutant meristems. Thus, transcriptional feedback regulation between *SEP1/2/3/4* paralogs and *AN/FA* (Fig 6O) ensures a critical *SEP* dosage to maintain *FA* and *AN* expression after the floral transition for accurate floral meristem patterning and floral organ differentiation. This model warrants further investigation using spatially resolved approaches to delineate feedback maintenance between *SEP* and *AN/FA* genes. While the spatiotemporal expression domains of *TM5* [46], *TM29* [47], *J2/SLMBP21* [59], *AN* [19], and *FA* [16] in floral meristems have been characterized by in situ hybridization experiments at tissue level individually, examining how these patterns are modified in *sep* and *an/fa* mutant backgrounds at cellular resolution will provide additional mechanistic insights.

### Gene dosage effects maintain *SEP* redundancy but constrain functional divergence

Multiple duplication events led to the expansion of the *SEP* gene family and allowed *SEP* paralogs to functionally diverge and acquire new functions [25,34]. We show that *SEP* redundancy is conserved during the transition of meristems to floral identity, seen in reversion of floral meristems to vegetative identity in *sep1234q* mutants. Redundant *SEP* roles during floral meristem development have been described in other species [34,35], which indicates that the *SEP* function in guiding meristem maturation towards floral identity is evolutionary conserved. *SEP3*, which belongs to a conserved paralog subclade that is maintained as one copy per diploid genome in most dicot species, thus evolved the derived and more specialized function in floral organ identity. Nevertheless, the dosage sensitivity of *SEP* genes during inflorescence and flower development imposed a constraint to the functional divergence of the *SEP* family. The molecular basis that underlies incomplete divergence of *SEP* paralogs remains to be determined. Divergent expression patterns from *cis*-regulatory changes may have led to functional divergence without unbalancing *SEP* dosage, and expression atlas data for *SEP* genes [7,11] hints towards regulatory sequence evolution although more detailed analyses of cell type-specific expression patterns are required. Future analyses of non-coding sequences may reveal *cis*-regulatory regions and transcriptional enhancers that control spatiotemporal *SEP* expression patterns underlying both divergent and redundant functions. In summary, our work demonstrates that dosage sensitivity of paralogous genes can impose an evolutionary constraint to the capacity of gene families to fully diverge in function.

## Methods

### Plant material and growth conditions

Wild-type seed for the *S. lycopersicum* cv. M82 (LA3475) and *S. lycopersicum* cv. Sweet-100 double-determinate [42] were from our own stocks. Mutant seed for *j2*, *ej2* and *lin* single and combinatorial mutant combinations in the M82 background were from our own stocks [14]. For phenotypic analyses, seeds were germinated on soil in 96-well plastic trays and grown under long-day conditions (16-h light/8-h dark) in a greenhouse supplemented with artificial light from LED panels (~250 μmol m^−2^ s^−1^), oscillating day-night temperature (25 °C day/20 °C night), and relative humidity of 50%–60%. Plants were grown in 5 L pots (2 per pot) under standard fertilizer regime and drip irrigation. For meristem analyses, seeds were pre-germinated on moist filter paper at 28 °C in the dark for 72 hours. Germinated seedlings with similar radicle length were transferred to soil in 96-well plastic flats and grown in the greenhouse on flooding tables.

### Plant phenotyping

To quantify flowering time, the number of leaves before the emergence of a floral meristem was counted. A minimum of nine plants was included for each of the genotypes. Additionally, the number of seedlings in vegetative, transition and floral meristem stage was assessed for each of the genotypes 20 days after sowing in a minimum of 22 plants per genotype. To quantify the number of flowers per inflorescence, flowers and floral buds were counted on five inflorescences per plant (excluding the first inflorescences) and 8 replicate plants per genotype.

Inflorescence branching was quantified by counting the number of branch points in five inflorescences per plant and eight replicate plants per genotype. Whenever that number exceeded 22 branching points, the inflorescence was considered branched but “uncountable”. All phenotyping experiments were performed for all the genotypes in parallel on a single day.

### Stereoscopy and photography

Seedlings grown in 96-well trays were harvested individually and all primordia except for the last two were removed by hand or with a needle. Z-stack images of developing meristems were acquired in different maturation stages spanning from vegetative to inflorescence using a Leica M205 FCA stereomicroscope with a Leica 10450028 objective connected to a Leica DFC7000T lamp (Leica Microsystems, Wetzlar, Germany). Photographs of inflorescences were taken using either an Olympus OM-D camera with an ED 14–42 mm f/3.5–5.6 EZ objective or an iPhone11 equipped with a 12 MP wide-angle camera.

### CRISPR-Cas

CRISPR–Cas9 mutagenesis in tomato was performed as previously described [60–62]. Briefly, guide RNAs (gRNAs) were designed using CRISPOR [63] and the Sweet-100v.2.0 reference genome [42]. Binary vectors for CRISPR-Cas mutagenesis were assembled using the Golden Gate cloning system as previously described [60,64,65]. The gRNA target sequences (gRNAs) were amplified using KOD Hot Start DNA Polymerase (Merck Millipore) using target-specific forward primer, a universal reverse primer and the pICH86966::AtU6p::sgRNA vector (Addgene 46966) as template. The gRNAs were combined with the AtU6p promoter in Level1 vectors, and assembled with Nos::NptII::ocs (addgene #51144) and SlUbi::SpCas9-P2A-GFP::nos [60] in Level2 binary vectors. Plasmids were verified by Sanger sequencing and transformed into *Agrobacterium tumefaciens* (AGL-1) by electroporation using a Bio-Rad GenePulser II (1 mm cuvettes, 1.8 kV, 25 µF, 200 Ω, ~5 ms pulse). Constructs were transformed into the double-determinate cherry tomato cultivar Sweet-100 or M82 by *Agrobacterium tumefaciens*-mediated transformation as previously described. CRISPR–Cas9 editing was verified in the T0 generation by genotyping, Sanger sequencing or amplicon deep sequencing as described [60], and transgene-free mutant plants were isolated in the T1. *J2*, *EJ2* and *LIN* were targeted simultaneously with a multiplex construct containing two gRNAs per gene. *TM5* and *TM29* were targeted with two independent constructs with two gRNA per gene, and mutations were combined by crossing T1 or T2 individuals. *FA* was targeted with three gRNA sequences. All gRNAs used in this study are listed in S12 Table.

### Genotyping

Frozen leaf or cotyledon tissue was homogenized with two metal beads at a frequency of 18–20 Hz for 1 min in a mix mill. Genomic DNA was extracted using extraction buffer (0.1 M Tris-HCl pH 9.5, 0.25 M KCl, 0.01 M EDTA) by vigorous vortexing. Cell extracts were transferred to new multi-well plate, incubated at 95 °C for 10 min, and subsequently cooled at 4 °C for 5 min. Equal volume of 3% (w/v) BSA was added to the lysate, mixed vigorously by vortexing, and centrifuged at 3,700 rpm for 15 min. Target regions were amplified with gene-specific primers and products were separated on agarose gels. For CAPS analyses, products were digested using restriction enzymes (NEB). For Sanger sequencing, PCR products were purified with ExoSap (Thermo). All primer sequences and genotyping information are listed in S13 Table.

### Amplicon deep sequencing

Genetic sector analyses were performed using amplicon deep sequencing as previously described [60,66]. Briefly, gDNA was extracted from individual flowers and gRNA target regions were amplified using gene-specific primers (S13 Table) with common adapter sequences. Gene-specific amplicons were diluted 10-fold in H_2_O and used as template for the addition of sample-specific indexes using adapter primers with unique barcodes as described before [42,66]. Equal volumes of each indexed amplicon reaction were pooled and subsequently purified using SPRIselect beads (Beckman Coulter). A single Illumina sequencing library was prepared using the xGen DNA Library Prep MC Kit (Integrated DNA Technologies) and sequenced on the MiSeq System (Illumina) at the Genome Technologies Facility (GTF) at UNIL. Raw reads were analyzed using the S100 reference genome [42,60] and a previously published pipeline [60] based on Trimmomatic (v0.39) [67], FASTX-Toolkit (v0.0.14) [68], PEAR (v0.9.6), HISAT2 (v2.2.1) [69], SAMtools (v.1.17) [70], SMAP [71,72], and MAFFT (v7.505) [73], retaining haplotypes with a minimum frequency of 5%. Haplotypes were categorized in “WT” (no insertions or deletions), “in-frame” (total InDel size a multiple of three or zero), “out-of-frame” (total InDel size not a multiple of three nor zero). Haplotype frequencies and sequences were visualized in R [74,75] and Benchling [76], respectively.

### Cis-regulatory sequence analyses

Accessible chromatin regions (ACRs) were identified using published ATAC-seq data [77]. Binding site for J2 were identified using published J2 ChIP-seq data [43]. Conserved non-coding sequences (CNS) were retrieved from the conservatory database [78]. MADS-box transcription factor binding sites were predicted on the SL4.0 reference genome [79] by FIMO/MEME Suite [80] using default parameters and the non-redundant set of profiles for MADS box factors from the JASPAR CORE database (release 2020) [45] (S14 Table). Data was visualized in jbrowse2 [81].

### Meristem dissection, RNA extraction, library preparation and sequencing

Thirteen days after sowing, three representative seedlings per 96-well flat were dissected to determine the average developmental stage of the seedling population. When representative seedlings had reached the transition or floral meristem stage of meristem maturation [7], seedings with similar number of leaves were rapidly harvested starting at 13:00 hours for a maximum of 2 hours to minimize circadian effects. After removal of leaves, shoot apices were fixed in ice-cold acetone and vacuum infiltrated for 45 min at 4 °C. Meristems were micro-dissected using a sharp syringe needle under a Leica MS5 stereomicroscope connected to a Leica CLS 100× lamp (Leica Microsystems, Wetzlar, Germany). Dissected meristems of the same genotype and morphological stage were pooled in 2 ml round-bottom RNAse-free Eppendorf tubes (Eppendorf, Hamburg, Germany) filled with ice-cold acetone, and stored at −70 °C. RNA was extracted from pools of 18–38 meristems and three replicate pools per genotype-stage combination using the Arcturus PicoPure RNA isolation kit (Applied Biosystems) according to the manufacturer protocol with minor modifications. Volumes of extraction buffer and 70% EtOH were increased 2.2-fold and RNA was treated with DNAse to remove contaminating gDNA using the RNAse-Free DNase kit (Qiagen). Sequencing libraries were prepared from 300 ng total RNA as starting material at the Genomic Technologies Facility (GTF) of the University of Lausanne. Indexed libraries were prepared using the TruSeq Stranded mRNA Library Prep kit from Illumina according to the manufacturer’s instructions. Fragment size and concentration were assessed with a Bioanalyzer. Libraries were sequenced on two Illumina NovaSeq6000 lanes to produce between 15,819,728 and 22,075,610 SR100 reads per sample (S15 Table).

### Read quality control and alignment

Read quality of raw reads was assessed using the FastQC (v0.11.7) [82]. Illumina TruSeq adaptors were trimmed with Trimmomatic (v0.36; with parameters SE -phred33 ILLUMINACLIP:TruSeq3-SE.fa:2:30:10 LEADING:3 TRAILING:3 SLIDINGWINDOW:4:15 MINLEN:36) [67]. Trimmed reads were aligned to the SL4.0 reference genome [79] using STAR (v2.7.8a; with parameters--runMode alignReads --outFilterType BySJout --outFilterMultimapNmax 20 --outMultimapperOrder Random --alignSJoverhangMin 8 --alignSJDBoverhangMin 1 --alignIntronMin 20 --alignIntronMax 1000000 --alignMatesGapMax 1000000 --outSAMtype BAM Unsorted) [83]. Alignments were sorted and indexed using SAMtools (v.1.17) [70] and gene expression was quantified as unique reads aligned to the SL4.0 gene annotation (ITAG4.0) [79] using HTSeq-count (v.0.11.2; with parameters --format = bam --order = pos --stranded = no --type = exon --idattr = Parent) [84].

### Differential expression (DE) analyses and identification of non-additive expression pattern

Transcriptomic data were processed using the edgeR (v4.4.2) [85] and limma (v3.62.2) [86] packages for differential expression analysis. Synergistic expression patterns were identified following the analysis framework described by Schrode and colleagues [39]. In brief, lowly expressed genes were filtered retaining only those with ≥10 reads in at least 2 samples. Counts were normalized using the trimmed means of *M*-values (TMM) method and transformed into log2 counts per million (logCPM) with the voom function() which also estimates mean-variance relationships and assigns precision weights for downstream linear modelling [39,86]. For each genotype (e.g., WT, *j2, ej2, j2ej2)*, a design matrix was constructed, and gene-wise linear models were fitted using the lmFit() function. For each set of singles and corresponding double mutant contrasts were defined as follows:

- Additive expectation = (*j2* − WT) + (*ej2* − WT)
- Combinatorial effect = (*j2ej2* − WT)
- Synergy (interaction) = (*j2ej2* − WT) − [(*j2* − WT) + (*ej2* − WT)]

For each gene, moderated *t*-statistics were calculated using the empirical Bayes procedure implemented in eBayes(), shrinking gene-wise variance estimates toward a common prior, stabilizing the standard errors (SE). Statistical significance was defined as genes with 95% confidence intervals excluding zero after Benjamini–Hochberg (BH) multiple-testing correction (FDR < 0.05). Each logFC estimate was tested as LogFC ± 1.96 × SE. Based on the calculated significance, genes were then classified as:

- Non-additive: when the synergy contrast was significant (FDR < 0.05). It can be:
  - Synergistic: when the double mutant effect exceeded the additive expectation in the same direction (i.e., more up or more down than predicted).
  - Less-than-additive: when the double mutant effect was weaker than expected.
- Additive when the double mutant was significantly different from control (FDR < 0.05) but the synergy contrast was not significant.

To further evaluate the robustness of synergy detection, the proportion of non-null hypotheses (*π*₁) across all synergy tests was estimated using the Storey–Tibshirani qvalue method [87]. Median standard error (SE) of contrasts was used to estimate the probability of detecting deviations from additivity at varying effect sizes and sample numbers [39]. Differentially expressed genes (DEGs) per genotype (single versus control or double versus control) were determined with a threshold of |log2FC| ≥ 0.58 (~1.5-fold change) and FDR < 0.01.

Multidimensional scaling (MDS) analyses were conducted using CPM values on: all expressed genes, 2,583 genes dynamically expressed during meristem maturation [15] and in 227 transition marker and 241 floral meristem marker genes, in the transition and floral stage datasets, respectively [14]. The samples linej2_TM_3 and M82_FM_1 were excluded from the differential gene expression analyses due to outlier behavior in the transition marker and floral marker MDS analyses (S2 Fig). Gene-normalized z-scores were calculated with the formula *z* = (*x* − *μ*)/*σ*, where *x* is the CPM value for a specific gene, *μ* is the mean of CPM values across samples, and *σ* is the standard deviation. All data visualization and statistical analyses were conducted in R [74,75]. Gene-normalized *z*-scores were plotted as heatmaps using pheatmap (v.1.10.12) [88] and dotplots using ggplot2 package (v3.5.1) [89]. Counts per million (CPM) were plotted in dotplots using ggplot2 (v3.5.1) [89]. Overlaps of DEGs were visualized using the eulerr package (v7.0.2) [90].

### RNA-seq analysis *sep1234q*, *fa, an*, and *sep4t* inflorescence tissue

Meristem tissue from *sep1234q* and *fa* mutants for mRNA extraction was collected at 13:00 hrs from mature plants grown in a greenhouse. Per genotype, three individual plants served as biological replicates, three inflorescences (~5 mm) were pooled per replicate, and flash-frozen in liquid nitrogen. Total RNA was extracted using the RNeasy Plant Mini Kit (QIAGEN) following the manufacturer’s protocol and assessed for quality using a Bioanalyzer. Sequencing libraries were prepared from 100 ng of total RNA at the Genomic Technologies Facility (GTF), University of Lausanne. In brief, indexed libraries were generated using the WATCHMAKER mRNA Library Prep Kit (Watchmaker Genomics) according to the manufacturer’s instructions, and library fragment size distribution and concentration were evaluated using a Bioanalyzer. Libraries were sequenced on two Element AVITI lanes, yielding between 17,145,097 and 20,335,750 paired-end 150 bp reads per sample. Meristem tissue from *sep4-triple* (*sep4t*; *j2 ej2 lin*) and *an* mutants for mRNA extraction was collected at 12:00 hrs from mature plants grown in a greenhouse. Per genotype, two individual plants served as biological replicates, three inflorescences (~5 mm) were pooled per replicate, and flash-frozen in liquid nitrogen. Total RNA was extracted using the RNeasy Plant Mini Kit (QIAGEN) following the manufacturer’s protocol and assessed for quality using a Bioanalyzer 2100 (Agilent). Libraries were prepared from 100 ng of total RNA at the Genome Center of Cold Spring Harbor Laboratories, Cold Spring Harbor, using the Kapa mRNA HyperPrep Kit (Kapa Biosystems) according to the manufacturer’s instructions. Libraries were sequenced on one NextSeq500 lane, yielding between 22′916′312 and 25′812′432 paired-end 75 bp reads per sample.

Read quality controls, alignments and counts were produced as described in the previous section. Lowly expressed genes were discarded by retaining only genes with ≥10 counts in at least 3 samples. Differential expression analysis and variance-stabilizing transformation (VST) was conducted in DESeq2 (v1.46.0) [91]. The transformed expression matrix was corrected for batch effects (WT samples [7] versus mutant (*sep1234q* and *fa*) samples) using the removeBatchEffect function from the limma (v3.62.2) [86]. Estimation of developmental stage was conducted using real-age prediction from transcriptome staging on reference (RAPToR, v1.2.0) [52]. A reference model was built on the staged WT meristem data using the functions ge_im() (formula = X ~ s(time, bs = “ts”, k = 5), nc = 3) and make_ref() (n.inter = 100). The WT reference model was used to stage mutant samples using the ae() function. Correlation profiles and stage estimates were visualized with plot() and plot_cor(), respectively, and mean stage estimates in ggplot2 (v3.5.2).

### Gene ontology enrichment analysis

Gene ontology enrichment analyses for biological processes were performed among differentially expressed genes using a functional annotation database for the ITAG4.0 tomato genome from PLAZA [92] and the ClusterProfiler package [93] with a pvalueCutoff = 0.05 and pAdjustMethod = BH. All annotated genes in the tomato genome (ITAG4.0) were used as background.

### Phylogenetic analysis

To reconstruct the phylogenetic tree of the SEP family across angiosperm species, a total of 22 proteomes were collected from the Orthologous MAtrix (OMA; release 12/2024) [48] database and the Conservatory CNS project [78] (S10 Table). Splice variants were removed to only retain primary protein isoforms and reduce redundancy. Hierarchical Orthologous Groups (HOGs, gene families) were obtained using FastOMA (v1.2.0) [94] and a species tree from the NCBI Taxonomy Database [95] as the guide phylogeny (S2 Data). The SEP and AGL6 gene families were identified using the proteins from *S. lycopersicum* and *A. thaliana* (S9 Table). The 135 protein sequences of this gene family were aligned with MAFFT (v7.505) [73], MUSCLE (v3.8.1551) [96] and Kalign (v3.3.2) [97] using default parameters. Resulting alignments were combined to a consistency-based alignment using T-Coffee (v13.46.0.919e8c6b) [98,99]. The maximum likelihood (ML) tree was inferred using IQ-TREE (v1.6.12) with 1,000 ultrafast bootstrap replicates [100]. This tree was pruned using the ETE4 (Environment for Tree Exploration) [101] toolkit to include only representatives of the Solanaceae family and Arabidopsis. To identify gene duplications, the gene trees were reconciled with the species tree via the species-overlap method [102] using the ETE4 toolkit.

## Supporting information

S1 Fig The *SEP4* paralogs *J2*, *EJ2*, and *LIN* have unequal contribution to inflorescence development. **(A)** Normalized read counts (transcripts per million, TPM) for the tomato *SEP4* genes *J2*, *EJ2*, *LIN*, and *RIN* in different tissues and meristem stages (EVM, early vegetative; MVM, middle vegetative; LVM, late vegetative; TM, transition; FM, floral; SIM, sympodial inflorescence; SYM, sympodial shoot meristem). **(B)** Schematic representation of *J2* (top), *EJ2* (middle), and *LIN* (bottom) with guide RNAs (gRNA) target positions indicated by arrowheads. Blue boxes represent exons and gray boxes represent UTRs (untranslated regions). The Cas9 cleavage sites for gRNAs are indicated with arrowheads. Mutant allele sequences identified by Sanger sequencing are shown with mutated nucleotide positions highlighted with red background. The Cas9 cleavage sites for guide RNAs are indicated with arrowheads and PAMs are marked in green. PAM, protospacer adjacent motif. **(C)** Stereomicroscopy images of a *j2* (left), *ej2* (middle), and *lin* (right) single mutant shoot apices with developing primary inflorescences. **(D)** Images of detached inflorescences of the *j2* (left), *ej2* (middle), and *lin* (right) single mutant. **(E)** Images of detached fruits with calyx of the WT (top), *j2* (middle) and *ej2* (bottom). Note the elongated sepals in *ej2*. **(F)** Images of inflorescences and hand-picked fruits of the WT and *j2*. Note the lack of fruit abscission at the pedicel in *j2*. **(G)** Detached inflorescences from the M82 wild-type (WT^M82^) and the mutant collection for *j2*, *ej2*, and *lin* single and double mutants in the tomato cultivar M82. Scale bars indicate 100 µm (C) and 1 cm (D-G). The data underlying this figure can be found in S1 Data.
(TIFF)

S2 Fig Multidimensional scaling (MDS) plots for gene expression profiles in transition and floral meristem samples. **(A)** MDS plots based on normalized counts for all expressed genes (left), genes dynamically expressed across stages of meristem maturation (middle), and transition stage marker genes (right) in all transition meristem (TM) samples. The outlier sample linej2_TM_3 is highlighted in magenta. **(B)** MDS plots as in (A) for transition meristem (TM) samples excluding linej2_TM_3 outlier sample. **(C)** MDS plots based on normalized counts for all expressed genes (left), genes dynamically expressed across stages of meristem maturation (middle), and floral stage marker genes (right) in all floral meristem (FM) samples. The outlier sample M82_FM_1 is highlighted in magenta. **(D)** MDS plots as in (C) for floral meristem (FM) samples excluding M82_FM_1 outlier sample. The data underlying this figure can be found in S1 Data.
(TIFF)

S3 Fig Phylogenetic analysis of the SEPALLATA MADS-box proteins across angiosperms. **(A)** Maximum-likelihood tree of SEP proteins in different angiosperm species. SEP1/2, SEP3, SEP4, and LOFSEP subclades are highlighted in different shades of purple, and the AGL6 sister clade as collapsed outgroup in yellow. Tomato and Arabidopsis genes are labeled in red and green font, respectively. Numbers at nodes represent bootstrap support values from 1,000 replicates. Branch lengths are proportional to the number of amino acid substitutions and are displayed for tomato and Arabidopsis proteins at the branch endpoints. Branches in green represent duplication events. The scale indicates the average number of substitutions per site. **(B)** Species tree with *SEP* gene counts across distinct subclades for angiosperm species shown in the phylogenetic tree in (A). Note that gene content of tetraploid *N. tabacum* is halved to allow comparisons with the other (diploid) genomes.
(TIFF)

S4 Fig Mutations in the *SEPALLATA1/2* paralogs *TM5* and *TM29* lead to defects in floral organ development. **(A)** Normalized read counts (transcripts per million, TPM) for *TM5* and *TM29* in different tissues and meristem stages (EVM, early vegetative; MVM, middle vegetative; LVM, late vegetative; TM, transition; FM, floral; SIM, sympodial inflorescence; SYM, sympodial shoot meristem). **(B)** Schematic representation of *TM5* (top) and *TM29* (bottom) with guide RNAs (gRNA) target positions indicated by arrowheads. Blue boxes represent exons and gray boxes represent UTRs (untranslated regions). The Cas9 cleavage sites for gRNAs are indicated with arrowheads. Mutant allele sequences identified by Sanger sequencing are shown with mutated nucleotide positions highlighted with red background. The Cas9 cleavage sites for guide RNAs are indicated with arrowheads and PAMs are marked in green. PAM, protospacer adjacent motif. **(C)** Photographs of detached inflorescences from the *tm29-1* and *tm5-1* single mutants. **(D, E)**, Detached inflorescences (D) and flowers (E) from three independent *tm5* mutant alleles. Note the outward-bending anthercone in *tm5* mutants. **(F)** Fruits of the WT and two independent *tm5* mutant alleles. Note the reduced seed number and size in *tm5* mutants. **(G)** Image of a representative inflorescence from the *tm5 tm29* double mutant with leaf-like floral organs. **(H)** Macroscopic image of a *tm5 tm29* flower with a developing pseudofruit (left) and an opened pseudofruit showing two locules filled with leaf-like organs. **(I)** Stereomicroscope images of developing carpels of the WT and *tm5 tm29* double mutant. Note that the *tm5 tm29* ovary in the image on the right has been opened to expose vegetative organs. **(J)** Stereomicroscope image of an *tm5 tm29 j2 triple* mutant flower. Note the emerging inflorescence with flower buds at the center of the flower. **(K)** Macroscopic image of a *tm5 tm29 j2* flower. Note the vegetative outer organs and the inflorescence in the center of the flower. **(L)** Model depicting the level of *SEP* gene redundancy during floral organ development in tomato. (1) *EJ2* activity specifies sepal identity: elongated sepals are observed in *ej2* single mutants but not in other *sep* single mutants, and *ej2* sepal defects are not enhanced in *ej2 sep* combinatorial mutants. (2) *TM5* and *TM29* act redundantly to specify petal identity: *tm5* and *tm29* single mutants develop partly fused petals, *tm5 tm29* double mutants develop leaf-like petals, and *tm5 tm29 sep4* combinations did not show additional petal defects. (3) *TM5* and *TM29* act redundantly to specify stamen identity with a larger contribution from *TM5*: *tm5* single and *tm5 tm29* double mutants develop curled anthers and leaf-like stamen, respectively. (4) *TM5*, *TM29*, *J2*, and *EJ2* act redundantly to specify carpel identity with a larger contribution from *TM5* and *TM29*: *tm5 tm29* double mutants develop parthenocarpic fruits filled with leaf-like organs, while carpels are transformed into ectopic inflorescences in *tm5 tm29 j2* and *tm5 tm29 ej2* mutants. Note that the impact of *LIN* on floral organ identity has not been investigated in this study. Scale bars: 1 cm in (C–H), (K) and 1 mm in (I–J). The data underlying this figure can be found in S1 Data.
(TIFF)

S5 Fig Reductions of *SEP1/2/3/4* activity and *FALSIFLORA* lead to floral organ defects and meristem overproliferation. **(A, B)** Images of *tm5 j2 ej2* and *tm29 j2 ej2* inflorescences (A) and dissected flowers (B). Se, sepals; pe, petals, st, stamen, ca, carpel. **(C)** Normalized read counts (transcripts per million, TPM) for *AN* and *FA* in different tissues and meristem stages (EVM, early vegetative; MVM, middle vegetative; LVM, late vegetative; TM, transition; FM, floral; SIM, sympodial inflorescence; SYM, sympodial shoot meristem). **(D)** Schematic representation of *FA* with guide RNAs (gRNA) target positions indicated by arrowheads. Purple boxes represent exons and green boxes represent UTRs (untranslated regions). Note that the SL4.0 reference annotation has been manually corrected. The Cas9 cleavage sites for gRNAs are indicated with arrowheads. Mutant allele sequences (identified by Sanger) are shown with mutated nucleotide positions highlighted in red background. The Cas9 cleavage sites for guide RNAs are indicated with arrowheads and PAMs are marked in green. PAM, protospacer adjacent motif. Note that *fa-6* and *fa-8* are biallelic and chimeric plants, respectively. **(E)** Images of inflorescences from the Sweet-100 WT (WT^S100^) and six independent first-generation (T0) *fa*-CRISPR (*fa*^*CR*^) transgenics. **(F)** Stereomicroscope images of inflorescence tissue from the *tm5 tm29 j2 ej2* quadruple mutant and a homozygous *fa-1* T1 plant. **(G)** Images of inflorescences of the WT and a f*a-1/+* heterozygote. **(H)** Images of inflorescences of the WT and different sectors of a *faCR* X WT F1 individual. Floral organ defects are labeled with magenta asterisks. Note the cauliflower inflorescence on the bottom-right. **(I)** Haplotype sequences at gRNA1/2 target (top) and gRNA3 target (bottom) are shown with mutated nucleotide positions highlighted in red background. The Cas9 cleavage sites for guide RNAs are indicated with arrowheads and PAMs are marked in green. PAM, protospacer adjacent motif. Scale bars: 1 cm in (A–B), (E), (G–H) and 500 µm in (F). The data underlying this figure can be found in S1 Data.
(TIFF)

S6 Fig Developmental stage prediction of *fa* and *sep1234q* mutant meristem tissue using RAPToR. **(A, B)** Correlation profiles for staged WT meristem (A) and *fa* and *sep1234q* mutant meristem (B) samples. Red bars indicate estimation confidence intervals, and black dotted lines indicate the 95% interval of bootstrap correlation with the reference. The sample age estimate is displayed below the interval. Estimated reference age is indicated in red font. **(C)** Developmental stage estimation for staged WT meristem samples and *fa* and *sep1234q* mutant meristem. Error bars indicate lower and upper bounds of the estimation confidence intervals, and vertical red lines indicate bootstrap estimates. The data underlying this figure can be found in S1 Data.
(TIFF)

S7 Fig Gene expression levels of *SEP1-4*, *AN* and *FA* in WT meristems and *an*, *fa*, *sep4t*, and *sep1234q* mutant inflorescence tissue. **(A, B)** Expression (normalized read counts) of *SEP1/2* (*TM29*), *SEP3* (*TM5*) and *SEP4* (*J2*, *EJ2*, *LIN*) genes, and *AN*/*FA* in staged WT meristems compared to *an* and *sep4* triple (*sep4t*; *j2 ej2 lin*) mutant inflorescence tissue (A), or compared to *fa* and *sep1234* quadruple (*sep1234q*; *tm5 tm29 j2 ej2*) mutant inflorescence tissue (B). EVM, early vegetative; MVM, middle vegetative; LVM, late vegetative; TM, transition; SIM, sympodial inflorescence; FM, floral meristem stage of meristem maturation). The data underlying this figure can be found in S1 Data.
(TIFF)

S1 Table 287 DEGs (FC > 1.5; FDR < 0.01) identified in transition meristem (TM) samples of the *j2 ej2* double mutant compared with the WT. The computed additive and nonadditive expression values in the double mutant and their class and direction are shown. Transcription factor family (TF family; PlantTFDB5.0), dynamic meristem expression (dynamic; Lemmon and colleagues, 2016, DOI: https://doi.org/10.1101/gr.207837.116) and J2 direct binding (J2_ChIPSeq; Wang and colleagues, 2023, DOI: https://doi.org/10.1093/plcell/koad065) is indicated.
(XLSX)

S2 Table 469 DEGs (FC > 1.5; FDR < 0.01) identified in transition meristem (TM) samples of the *lin j2* double mutant compared with the WT. The computed additive and nonadditive expression values in the double mutant and their class and direction are shown. Transcription factor family (TF family; PlantTFDB5.0), dynamic meristem expression (dynamic; Lemmon and colleagues, 2016, DOI: https://doi.org/10.1101/gr.207837.116) and J2 direct binding (J2_ChIPSeq; Wang and colleagues, 2023, DOI: https://doi.org/10.1093/plcell/koad065) is indicated.
(XLSX)

S3 Table 520 DEGs (FC > 1.5; FDR < 0.01) identified in transition meristem (TM) samples of the *lin ej2* double mutant compared with the WT. The computed additive and nonadditive expression values in the double mutant and their class and direction are shown. Transcription factor family (TF family; PlantTFDB5.0), dynamic meristem expression (dynamic; Lemmon and colleagues, 2016, DOI: https://doi.org/10.1101/gr.207837.116) and J2 direct binding (J2_ChIPSeq; Wang et al., 2023, DOI: https://doi.org/10.1093/plcell/koad065) is indicated.
(XLSX)

S4 Table 351 DEGs (FC > 1.5; FDR < 0.01) identified in floral meristem (FM) samples of the *j2 ej2* double mutant compared with the WT. The computed additive and nonadditive expression values in the double mutant and their class and direction are shown. Transcription factor family (TF family; PlantTFDB5.0), dynamic meristem expression (dynamic; Lemmon and colleagues, 2016, DOI: https://doi.org/10.1101/gr.207837.116) and J2 direct binding (J2_ChIPSeq; Wang and colleagues, 2023, DOI: https://doi.org/10.1093/plcell/koad065) is indicated.
(XLSX)

S5 Table 574 DEGs (FC > 1.5; FDR < 0.01) identified in floral meristem (FM) samples of the *lin j2* double mutant compared with the WT. The computed additive and nonadditive expression values in the double mutant and their class and direction are shown. Transcription factor family (TF family; PlantTFDB5.0), dynamic meristem expression (dynamic; Lemmon and colleagues, 2016, DOI: https://doi.org/10.1101/gr.207837.116) and J2 direct binding (J2_ChIPSeq; Wang and colleagues, 2023, DOI: https://doi.org/10.1093/plcell/koad065) is indicated.
(XLSX)

S6 Table 308 DEGs (FC > 1.5; FDR < 0.01) identified in floral meristem (FM) samples of the *lin ej2* double mutant compared with the WT. The computed additive and nonadditive expression values in the double mutant and their class and direction are shown. Transcription factor family (TF family; PlantTFDB5.0), dynamic meristem expression (dynamic; Lemmon and colleagues, 2016, DOI: https://doi.org/10.1101/gr.207837.116) and J2 direct binding (J2_ChIPSeq; Wang and colleagues, 2023, DOI: https://doi.org/10.1093/plcell/koad065) is indicated.
(XLSX)

S7 Table 68 genes differentially expressed (FC > 1.5; FDR < 0.05) in transition meristem (TM) samples of *j2ej2*, *linj2* and *linej2* compared with the WT. Transcription factor family (TF family; PlantTFDB5.0), dynamic meristem expression (dynamic; Lemmon and colleagues, 2016, DOI: https://doi.org/10.1101/gr.207837.116) and J2 direct binding (J2_ChIPSeq; Wang and colleagues, 2023, DOI: https://doi.org/10.1093/plcell/koad065) is indicated.
(XLSX)

S8 Table 104 genes differentially expressed (FC > 1.5; FDR < 0.01) in floral meristem (FM) samples of *j2ej2*, *linj2* and *linej2* compared with the WT. Transcription factor family (TF family; PlantTFDB5.0), dynamic meristem expression (dynamic; Lemmon and colleagues, 2016, DOI: https://doi.org/10.1101/gr.207837.116) and J2 direct binding (J2_ChIPSeq; Wang and colleagues, 2023, DOI: https://doi.org/10.1093/plcell/koad065) is indicated.
(XLSX)

S9 Table SEPALLATA proteins in *A. thaliana* and *S. lycopersicum* and their corresponding HOG category. (XLSX)

S10 Table SEPALLATA and AGL6 proteins from selected angiosperm species. (XLSX)

S11 Table *FA* haplotype frequencies quantified by amplicon deep sequencing. (XLSX)

S12 Table Sequences of gRNA target sequences used in this study. (XLSX)

S13 Table Primers used in this study. (XLSX)

S14 Table JASPAR CORE database matrix IDs for MADS box factors used in this study. (XLSX)

S15 Table Number of reads and mapping rates. (XLSX)

S1 Data Source data file containing numerical values for figures. (XLSX)

S2 Data NCBI Taxonomy Database species tree. (NWK)

## Abbreviations

ACR: accessible chromatin region
BH: Benjamini–Hochberg
ChIP-seq: Chromatin Immunoprecipitation-sequencing
CNS: conserved noncoding sequences
CREs: *cis*-regulatory elements
DEGs: differentially expressed genes
EMSAs: electrophoretic mobility shift assays
FM: floral meristem
GO: gene ontology
gRNAs: guide RNAs
GTF: Genome Technologies Facility
HOGs: hierarchical orthologous groups
MDS: multidimensional scaling
ML: maximum likelihood
PCA: principal component analyses
SE: standard errors
SIM: sympodial inflorescence meristem
TM: transition meristem
TMM: trimmed means of *M*-values
VM: vegetative meristem
VST: variance-stabilizing transformation
