# Supplementary material for: Incomplete functional divergence drives MADS-box gene synergy during floral development in tomato
Source: PLoS Biol. 2026 Aug 18;24(8):e3003946. doi: 10.1371/journal.pbio.3003946 (PMC13506103; doi:10.1371/journal.pbio.3003946)
Supplement: Supplementary file 1 [file pbio.3003946.s027.pdf]

We thank the three reviewers and the editors for their time and effort in reviewing our manuscript. We appreciate their constructive feedback and the opportunity to respond to their comments with a revised version of the manuscript. Please find below our detailed responses to all comments and questions.

Editorial office:

Thank you for your patience while your manuscript "Incomplete functional divergence drives genetic synergy during floral development in tomato" was peer-reviewed at PLOS Biology. It has now been evaluated by the PLOS Biology editors, an Academic Editor with relevant expertise, and by three independent reviewers.

In light of the reviews, which you will find at the end of this email, we would like to invite you to revise the work to thoroughly address the reviewers' reports.

You will see that all three reviewers find the study interesting and worth pursuing, but they also raise several issues that would need to be addressed before we can consider the manuscript for publication. Reviewer #1 suggests adding a final overview figure illustrating the different levels of redundancy among the SEP genes, along with a few minor comments for clarity. Reviewer #2 thinks that performing in situ hybridization would spatially validate the main regulatory interactions, or at least existing data available in the literature could be discussed. In addition, this reviewer proposes adding a summary schematic (a GRN) highlighting the newly identified regulatory interactions to complement the final figure. Reviewer #3 recommends reframing the study, perhaps simply as "dissecting the genetic redundancy of the SEP genes and their target genes."

After discussing these comments with the Academic Editor and the rest of the team, we have decided to invite a revision that should address all the points raised by the reviewers. We agree with Reviewer #2 that additional in situ experiments would strengthen the paper, but if there are some already available in the literature, you may add them.

We appreciate the editorial comment and invitation to submit a revised version of this manuscript. In this revised version, we specifically addressed the following points mentioned by the editorial office:

1. We now provide an overview figure that illustrates the different levels of redundancy among SEP genes during floral organ development (**Figure S4L**).
2. We present a new RNA-seq dataset and global expression analyses for inflorescence tissue of the *anantha* and *sep4* triple (*j2 ej2 lin*) mutant. Together with additional analyses of the previously existing expression data, we now provide further support for the identified regulatory interactions (**Figure 4I-J** and **Figure S7**). In addition, we cite and discuss RNA in-situ hybridisation datasets

from the literature. Finally, we added a summary schematic to the final figure (**Fig. 6O**) to describe the regulatory interactions identified in this work

3. We made several changes throughout the manuscript including the title and abstract to tone down generalized statements about novelty, and now better frame our findings in the contexts of *SEP* gene redundancy.

These points and all other comments and questions raised by the reviewers are addressed in more detail below.

Reviewer #1:

Reviewer 1, comment 1:

In their manuscript entitled "Incomplete functional divergence drives genetic synergy during floral development in tomato", Gaarslev et al. aim to determine the reason for non-additive inflorescence phenotypes for single, double, and triple sep4-like mutants in tomato. They are specifically interested in the developmental genetic basis of differences in "synergistic" interactions between paralogous pairs, whereby loss-of-function mutations at two or more loci have a more dramatic phenotypic effect that mutations in the single mutants combined. To do this, the authors characterize inflorescence phenotypes and compare differentially expressed gene expression in falsiflora (fa) and sep1/2/3/4 mutant combinations. Results showed that j2:ej2 double mutants had more synergistic inflorescence phenotypes than lin:j2 or lin:ej2 double mutants, and that SEP1/2/3 are partially redundant with SEP4 genes in conferring floral organ identity/determinacy. The authors also identified ANANTHA (AN)and possibly FA as direct targets of SEP4-like proteins that is responsive to SEP4-like dosage effects. The manuscript is well written and logically laid out. Despite the Results being rather long, they were easy to read. The authors clearly conducted a lot of experimental work for this manuscript, and I found the analyses to be appropriate for the study.

Response: We thank this reviewer for their positive assessment and their interest in our efforts to dissect the genetic and molecular basis of synergistic interactions among *SEP4*-like genes during tomato inflorescence development. We are especially grateful for the comment regarding the extend of the experimental work and the quality of writing. We are also very grateful for the detailed and specific comments that helped us to substantially improve clarity, quality and accessibility of the manuscript.

Reviewer 1, comment 2

I know there are already quite a few main figures. However, given all this work, I wonder whether the authors would consider adding a final overview figure, illustrating the different levels of redundancy for the SEP genes. There was a nice one in Fig. 3a in Rodriguez-Leal et al. (2019) Nature Genetics 51: 786-792 that was on genetic compensation in tomato. Other than that, my comments are minor and outlined below.

Response: We thank the reviewer for this helpful suggestion. We agree that a schematic overview would facilitate the interpretation of the different levels of redundancy among SEP genes. In response, we have added a new panel to the figure (**Fig. S4L**) illustrating the different levels of *SEP* redundancy during floral organ development identified in this study. In addition, we added a final overview in the final main figure highlighting the identified regulatory interactions between *SEP* genes and

the *AN-FA* module (**Fig. 6O**). We believe that these new figure panels enhance the interpretation of our work and make our findings more accessible to readers.

In **Figure S4** we added the following panel and extended legend:

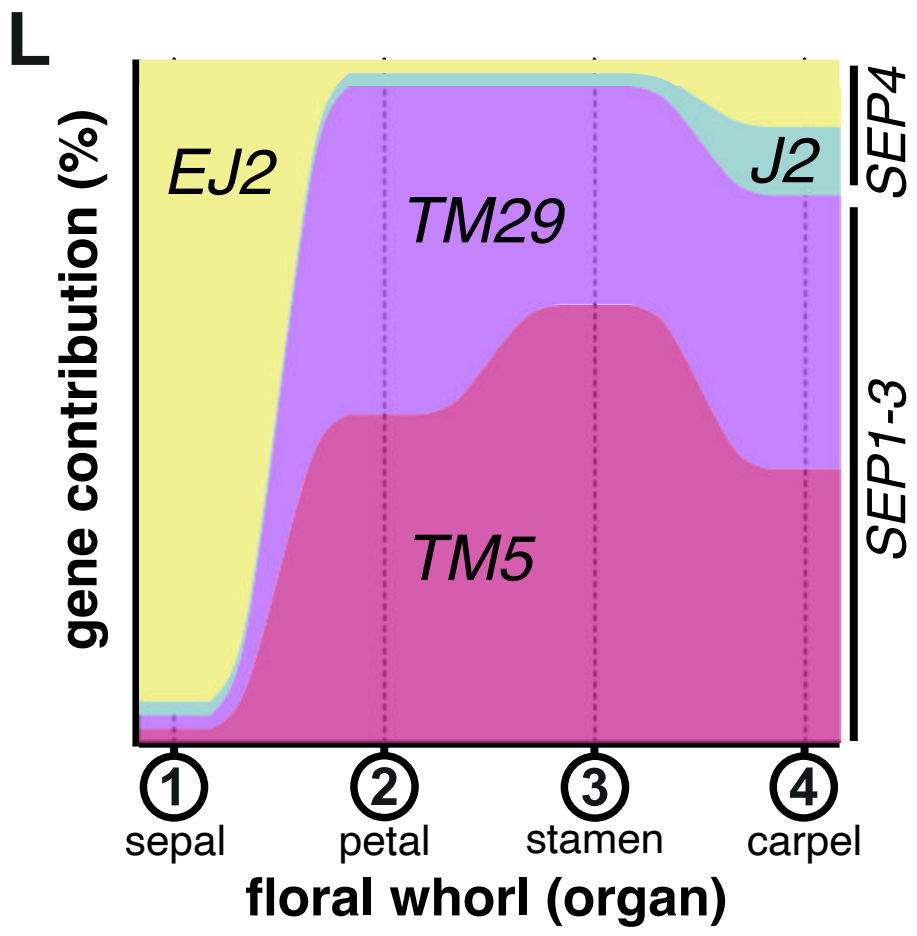

(**L**) Model depicting the level of *SEP* gene redundancy during floral organ development in tomato. (1) *EJ2* activity specifies sepal identity: elongated sepals are observed in *ej2* single mutants but not in other *sep* single mutants, and *ej2* sepal defects are not enhanced in *ej2 sep* combinatorial mutants. (2) *TM5* and *TM29* act redundantly to specify petal identity: *tm5* and *tm29* single mutants develop partly fused petals, *tm5 tm29* double mutants develop leaf-like petals, and *tm5 tm29 sep4* combinations did not show additional petal defects. (3) *TM5* and *TM29* act redundantly to specify stamen identity with a larger contribution from *TM5*: *tm5* single and *tm5 tm29* double mutants develop curled anthers and leaf-like stamen, respectively. (4) *TM5*, *TM29*, *J2*, and *EJ2* act redundantly to specify carpel identity with a larger contribution from *TM5* and *TM29*: *tm5 tm29* double mutants develop parthenocarpic fruits filled with leaf-like organs, while carpels are transformed into ectopic inflorescences in *tm5 tm29 j2* and *tm5 tm29 ej2* mutants. Note that the impact of *LIN* on floral organ identity has not been investigated in this study.

In **Figure 6** we added the following panel and extended legend:

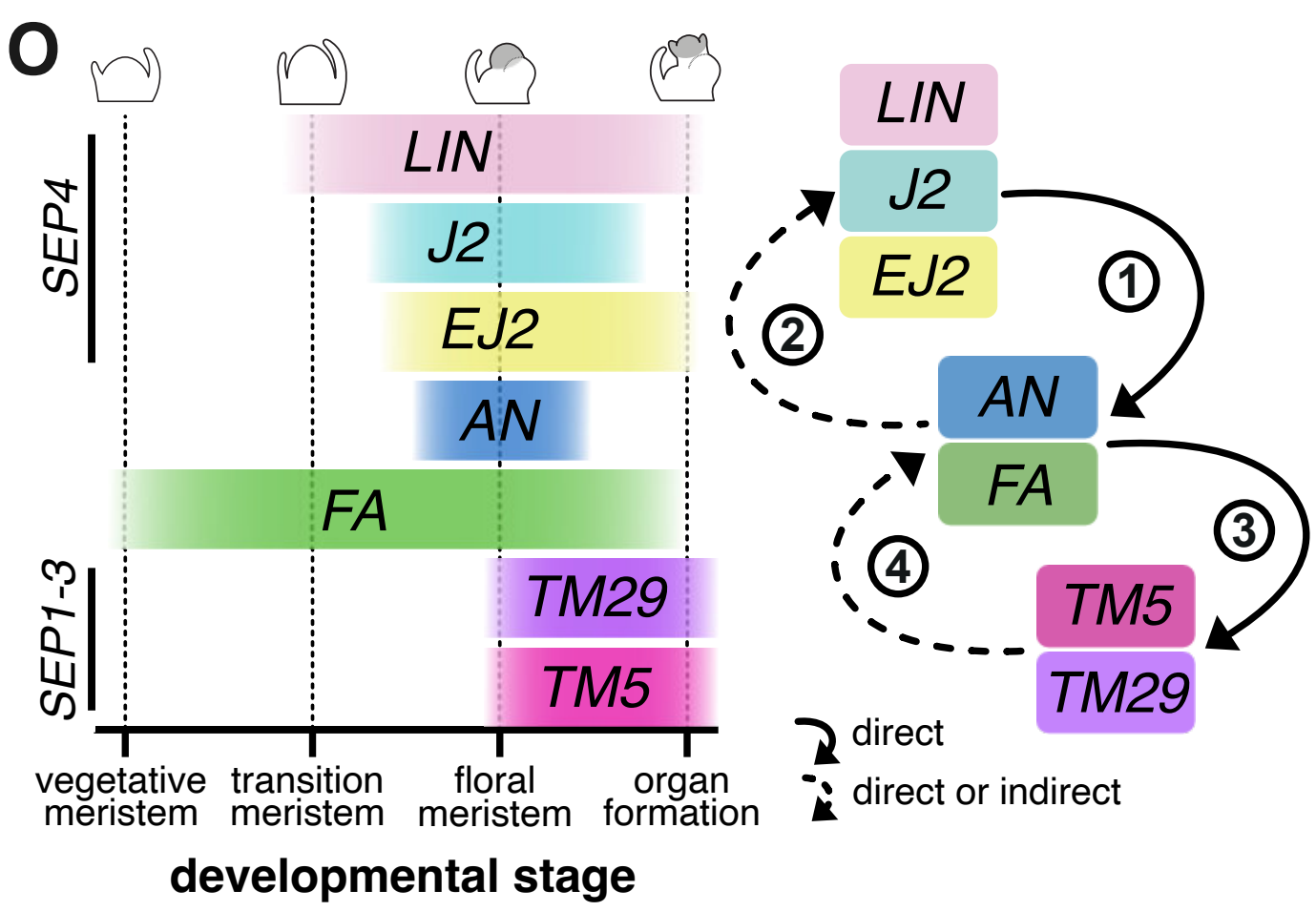

(**O**) Proposed regulatory feedback between *SEP* genes and *AN/FA*. Stage-specific expression patterns of *SEP1-4* and *AN/FA* (left) support a sequential, positive feedback between *SEP* genes and the *AN/FA* module (right): (1) At the transition from vegetative to floral meristem identity, *SEP4* genes redundantly activate *AN*, supported by *sep4*-triple and *an* cauliflower-like inflorescences, low *AN* expression in *sep4* single and combinatorial mutants, and J2 binding upstream of *AN*. (2) *AN/FA* positively feedbacks on

SEP4 to maintain floral meristem identity, supported by downregulation of *SEP4* genes in an meristems. (3) *AN/FA* activate *SEP1-3* genes in floral meristems to specify floral organ identity, supported by *SEP1-3* downregulation in *an* and *fa* meristems. (4) *SEP1-4* positively feedbacks on *FA* to maintain floral meristem identity, supported by low *FA* expression in *sep4t* and *sep1234q* meristems. Solid and dashed arrows mark regulation with support for direct (genetic analyses, RNA-seq, ChIP-seq) and indirect regulation (genetic analyses, RNA-seq), respectively.

Reviewer 1, comment 3

Line 51: should this be "transitional meristem"? I haven't encountered "transition meristem" before but maybe this is a tomato term.

Response: We thank the reviewer for the comment and agree that the term “transition meristem” can be misleading and may conflict with the nomenclature for the stages of meristem development in other species. We follow a specific definition of distinct developmental stages during tomato shoot apical meristem maturation, which was described by Park et al., 2011 (PMID: 22203998). We now better specify this in the text.

On page 2, we now write:

In tomato (*Solanum lycopersicum*) and other sympodial plants, the primary shoot apical meristem progresses from a vegetative meristem (VM) through the transition meristem (TM) and the floral meristem (FM) stage of meristem maturation [4], and terminates in the first flower of the multi-flowered inflorescence (**Figure 1A**).

Reviewer 1, comment 4

Line 60: should this say "In tomato mutants"? Or is this referring to natural variation in tomato?

Response: We thank the reviewer for this comment. A study by Lemmon et al., 2016 (PMID: 27821409) indeed demonstrated that also natural variation in inflorescence branching of tomato (and related nightshade species) can emerge from delays or accelerations in meristem maturation. Still, we agree that referring to mutants here is more accurate.

On page 2, we now write:

In tomato mutants, precocious expression programs result in premature termination of meristems and less complex inflorescences [10,11],

Reviewer 1, comment 5

Line 66: I think this should say Arabidopsis thaliana at first mention.

Response: Thank you for this comment. On page 3, we now write:

the ortholog of *Arabidopsis thaliana LEAFY* (*LFY*)

Reviewer 1, comment 6

Line 90: perhaps reword to "...expanded to include genes with E-class function,...)?

Response: We thank the reviewer for this suggestion. On page 3, we now write:

In the early 2000s, the ABC model was expanded to include genes with the E-class function,

Reviewer 1, comment 7

Line 102: given what you've already said, perhaps reword to "In addition to J2, EJ2 and LIN, the tomato genome encodes a fourth SEP4 paralog RIPENING INHIBITOR (RIN).

Response: We thank the reviewer for this comment. On page 4, now write:

In addition to *J2*, *EJ2*, *LIN*, tomato encodes a fourth *SEP4* paralog *RIPENING INHIBITOR* (*RIN*) [12].

Reviewer 1, comment 8

Line 128: do the j2 ej2 double mutants that produce flowers also show the single mutant phenotypes of loss of fruit abscission and elongated sepals? What about the other mutants?

Response: We thank the reviewer for this thoughtful question. It has been reported before that the *j2 ej2* double mutants indeed also display the single mutant phenotypes, i.e. loss of fruit abscission (from *j2*) and elongated sepals (from *ej2*) (see Figs 1E and S1C in Soyk et al., 2017, PMID: 28528644). In this study, we observed similar additive relationships in *lin j2* and *lin ej2* double mutants. The *lin j2* double mutant lacks fruit abscission zones (from *j2*) while *lin ej2* develops enlarged sepals (from e*j2*) (see images in **Figure S1G**). In addition, both *lin j2* and *lin ej2* develop elongated inflorescences from *lin* (see images and quantitative data in **Figure 2F-G**). We now explicitly describe these additive relationships between *J2*, *EJ2*, and *LIN* in the results section and refer to the relevant figures and literature.

On page 4, we now write:

In addition, *j2 ej2* double mutants produced ectopic inflorescence meristems, which resulted in strongly branched inflorescences on primary and sympodial shoots bearing flowers with elongated sepals and missing abscission zones [13] (**Figure 2A-C**).

On page 5 we now write:

In addition, *lin j2* lacked fruit abscission zones while *lin ej2* developed elongated sepals, reminiscent of the *j2* and *ej2* single mutants (**Figure S1G**).

Reviewer 1, comment 9

Lin 135: I'd suggest saying "between J2 and EJ2" rather than "J2/EJ2" since the latter usually denoted two genes as a unit.

Response: Thanks for this suggestion. We apologize for the inconsistency in using the appropriate nomenclature.

On page 5, we now write:

These observations indicate synergistic interactions between *LIN* and both *J2* and *EJ2* during inflorescence development, however, the effect was weaker when compared with synergy between *J2* and *EJ2* [13].

Reviewer 1, comment 10

Line 191: Rather than "DEGs unlikely explain", I suggest "DEGs are unlikely to explain".

Response: Thanks for this suggestion, on page 6 we now write:

On this basis, less-than-additive DEGs are unlikely to explain the synergistic inflorescence phenotypes that are only observed in the double mutants.

Reviewer 1, comment 11

Line 239: Maybe it is a journal requirement, but usually family and subfamily names are not in italics.

Response: Thanks for this comment. We now write Solanoideae and Solanaceae in non-italic type throughout the manuscript.

Reviewer 1, comment 12

Line 259: should be "floral organ specification" not "organs".

Response: Thanks for this comment, we corrected this typo on page 9.

Reviewer 1, comment 13

Line 285: what do you mean by "SEP4 is partially lost"? Do you mean SEP4 function?

Response: Apologies for this oversight. We indeed do not refer to gene loss (presence-absence variation) but to the loss of gene function. To clarify, we now write on page 9:

These results show that when *SEP4* function is partially lost in the absence of *SEP1/2/3* (in *tm5 tm29 j2* or *tm5 tm29 ej2*), floral meristems produce three whorls of vegetative organs.

Reviewer 1, comment 14

Line 342: "as a reference" rather than "as reference"?

Response: Thanks for this comment, we now write on page 11.

To estimate the developmental stage of *fa* and *sep1234q* meristem tissue, we applied an established framework [51] and staged mutant transcriptomes on the WT meristem maturation trajectory as a reference (**Figure S6** and **Methods**).

Reviewer 1, comment 15

Line 361: I think I'd say "indicating that synergistic effects declined with evolutionary distance" or "indicating that synergistic effects may generally decline...". The way it is worded now suggests a statement of fact about synergy, which I don't think the authors can declare with a single example.

Response: Thanks for the comment and apologize for overgeneralisation of our findings. We followed your specific text suggestion and now write on page 12:

Here, we found that *SEP4* synergy on gene expression programs underlying inflorescence branching is more pronounced between the direct paralogs *J2* and *EJ2* than with the distant paralog *LIN*. This pattern is consistent with paralog redundancy generally declining over evolutionary time, likely driven by divergence in coding sequences and gene expression patterns[53].

Reviewer 1, comment 16

Line 367: why "predetermined" and not just "determined"? To have made a decision does not necessarily mean steps have been taken toward the decided goal.

Response: Thanks for the comment, we fully agree and now write on page 12:

By integrating data from previous studies [42,43], we propose a model (**Figure 6O**) in which SEP4 paralogs directly activate *AN* expression by binding an enhancer element ~3.5 kbp upstream of *AN* when meristems transition to floral identity, a critical window in which inflorescence architecture is determined [6,13,14].

Reviewer 1, comment 17

Line 419: I'm curious as to what evidence is available to say that SEP3 is the ancestral paralogue since paralogues are by their very nature equidistant to each other in a phylogenetic sense. Is this based on synteny or does the SEP3 sequence better resemble what we think the ancestral (i.e. pre-duplication) SEP sequence looked like?

Response: We thank the reviewer for pointing out this ambiguity. We originally used “ancestral paralog” to indicate that *SEP3/TM5* belongs to a *SEP* paralog subclade that traces back to an ancestral duplication predating the divergence of Arabidopsis and tomato. However, we agree that this wording could be misunderstood as implying that *SEP3* represents the ancestral copy after duplication, which was not our intention. To avoid this ambiguity, we have revised the sentence to refer instead to *SEP3* as belonging to a conserved *SEP* paralog subclade that is retained as a single copy per diploid genome in most dicot species.

We now write on page 15:

SEP3, which belongs to a conserved paralog subclade that is maintained as one copy per diploid genome in most dicot species, thus evolved the derived and more specialized function in floral organ identity.

Reviewer 1, comment 18

Line 450-456 (and elsewhere): numbers below 11 are usually spelled out in full.

Response: Thanks for pointing this out, we now write on page 16:

A minimum of nine plants was included for each of the genotypes.

Inflorescence branching was quantified by counting the number of branch points in five inflorescences per plant and eight replicate plants per genotype.

Reviewer 1, comment 19

Line 470-473: perhaps remove one of the "Briefly"s.

Response: Thanks for this suggestion to improve the text, we now write on page 17:

The gRNA target sequences (gRNAs) were amplified using KOD Hot Start DNA Polymerase (Merck Millipore) using target-specific forward primer, a universal reverse primer and the pICH86966::AtU6p::sgRNA vector (Addgene 46966) as template.

Reviewer #2:

Reviewer 2, Comment 1:

Through phenotypic characterization of multiple SEP4 mutants (affecting the three MADS-box genes from the SEP4 family: LIN, J2, and EJ2), combined with gene expression analyses, this study establishes the relative importance of the various SEP4 genes in tomato flower development. It then focuses on several known SEP4 targets—key regulators—to deepen our understanding of the gene regulatory network (GRN) controlling flower development (involving AN, FA, and SEP1-3).

This manuscript presents a substantial body of high-quality data and analyses, with visually compelling figures. While some results are novel and exciting, others are more descriptive and confirmatory. Overall, the work represents a significant contribution to elucidating how flowers develop in tomato.

Response: We thank this reviewer for these very encouraging words, especially for pointing out the quality and novel aspects of our work. We are pleased that see that our phenotypic and gene expression analyses provide novel insight into the gene regulatory network underlying tomato flower development.

Reviewer 2, Comment 2:

The analysis of gene expression in the three double mutants affected in J2, EJ2, and LIN is particularly interesting. It not only identifies differentially expressed genes (DEGs) but also explores the nature of their regulation. A less-than-additive effect suggests that the first SEP mutation severely impairs the activity of the protein complex, rendering a second mutation less impactful—implying that the complex likely contains two SEP4 members. Conversely, strong synergy indicates redundancy, where one mutated SEP4 member can be almost fully compensated by another (and thus a distinct complex). To my knowledge, this is a rare and highly informative analysis. However, the study does not attempt to link these observations to the actual composition of heterotetrameric complexes or their regulatory signatures (e.g., CArG boxes) in the DEGs. It would be valuable to speculate in the discussion about the nature of these tetrameric complexes and their potential additional members (e.g., FUL/AP1 or SEP1-3 clades). Ideally, supporting these hypotheses with existing genomic data would further strengthen the work.

Response: We appreciate the reviewer's appreciation of our gene expression analyses in single and double *sepallata* mutants to explore non-additive patterns in gene expression. We agree that integrating the expression data with regulatory signatures of SEP containing complexes is an important future direction. We would like to emphasize that we already integrated a published J2 ChIP-seq dataset (by Wang et al., 2023, PMID:

36881857) in the first version of the manuscript, which helped to identify *ANANTHA* (tomato *UFO*), and potentially *FALSIFLORA* (tomato *LEAFY*), as direct target of the SEP4 factors J2 (see **Figs. 4H and 6K**). We also used MEME-FIMO to predict genome-wide CArG-boxes using frequency matrixes for Arabidopsis MADS-box proteins from the JASPAR database (see **Figs. 4H and 6K**) but we believe that these predictions on its own are not sufficient to make meaningful conclusions about functional MADS-box binding sites without additional experimental protein-DNA binding data. For these reasons, we refrained from a global integration of these CArG-box predictions with the different DEG categories.

The J2 ChIP-seq dataset by Wang et al. is currently the only *SEP* ChIP-seq dataset available in tomato. In response to this comment, we also analyzed a recently published DAP-seq dataset for the tomato MADS-box J2/MC and J2/FUL2 complexes (Jiang et al., 2025, PMID: 40827093). With this data we could not confirm the J2 binding site upstream of *ANANTHA* that we identified in the ChIP-Seq data by Wang et al., which is located in a regulatory elements that has been shown to affect *AN* activity when mutated by CRISPR (Lanctot et al., 2025, PMID: 39964724). We believe that this discrepancy between DAP-seq and ChIP-seq highlights the difficulty of reconstituting functional MADS-box tetramers *in-vitro* and the need to identify and interrogate MADS-box binding sites *in vivo*. Still, we now cite and discuss the DAP-seq data by Jiang et al in the discussion part of the manuscript.

We agree that the protein composition of the heterotetrameric *SEP* complexes that regulate meristem transitions and floral organogenesis is a highly relevant question in the scientific field of flower development. We now discuss the publications that were highlighted by this and other reviewers and speculate that SEP proteins interact with AP1/FUL-clade MADS-box factors during different stages of meristem maturation and floral organ formation. Our hypotheses are mainly based on mutants in the *AP1*-like gene *MACROCALYX* (*MC*) and the *FUL*-like genes *FRUITFUL2* (*FUL2*) and *MBP20*, which show different levels of defects in floral meristem maturation and floral organ identity. We describe this model in a new paragraph in the discussion.

On page 13, we now write:

An open question is which other MADS-box factors interact with SEP proteins to form heterotetrameric complexes that bind to the CArG-box target motifs upstream of *AN* and *FA*. We speculate that SEP proteins interact with AP1/FRUITFULL (FUL)-clade MADS-box proteins that are co-expressed in transition and floral meristems [6,10,54]. This model is supported by published yeast two-hybrid assays showing interactions between the AP1/FUL-like proteins MACROCALYX (MC) and FUL2, and the SEP proteins TM5, J2, EJ2, and RIN [54,55]. A recent study[54] reported that heterotetramers of J2 and either MC or FUL2 bind target DNA in

electrophoretic mobility shift assays (EMSAs). We revisited affinity purification-sequencing (DAP-seq) experiments with *in-vitro*-reconstituted heterotetramers from this study but did not find evidence for J2/MC or J2/FUL2 binding at the J2 target sites upstream of *AN* and *FA* identified in *in-vivo* meristem ChIP-seq data [42]. However, genetic evidence supports a role of *MC*, *FUL2*, and the *FUL*-like gene *MBP20* in meristem maturation and floral meristem identity. Combined loss of *FUL2* and *MBP20* function results in a flowering delay and moderately branched inflorescences that resemble those of weak *j2 ej2* mutants [54]. The *mc* mutant also flowers late but produces flowers with elongated sepals similar to *ej2* mutants [54,56]. However, in contrast to *ej2*, *mc* inflorescences revert to vegetative growth after the formation of few flowers and this vegetative reversion is strongly enhanced in *mc ful2 mbp20*, indicating redundant roles of *AP1/FUL* genes in conferring floral identity [54,56]. Together, these findings show that *AP1/FUL*-genes are involved in both guiding meristem transitions and specifying floral organ identity, paralleling the roles of *FA* and *SEP* genes described in this study. This dual function supports a model in which the floral transition and floral organogenesis represent a developmental continuum rather than distinct phases [57].

Reviewer 2, Comment 3:

Beyond SEP4 genes, this study also addresses a critical component of the GRN governing inflorescence development. The role of AN is especially intriguing, as the an single mutant phenocopies the j2 ej2 lin triple mutant. The data show that AN expression is regulated by the SEPs (important new result), but it remains unclear how much of the triple sep4 mutant phenotype can be attributed to this regulation.

Response: We thank the reviewer for this intriguing question. The *anantha* mutant indeed phenocopies the *j2 ej2 lin* (*sep4*-triple; *sep4t*) mutant during early stages of inflorescence development. Both mutants develop cauliflower-like inflorescences that consist of meristematic tissue, although in our hands the *sep4t* mutant can produce very few fertile flowers depending on the season. Interestingly, a similar phenomenon had been reported for the genetic equivalent in petunia (*fbp4 fbp9 fbp23*) by Morel et al., 2019 (PMID: 31591161). As the reviewer points out, we proposed that *AN* expression is directly activated by the *SEP4* genes but did not assess if *ANANTHA* transcript levels change in *j2 ej2 lin* triple mutants. Low expression of *AN* in *sep4t* mutants could explain their *an*-like phenotype.

To address this question, we conducted additional RNA-seq analyses on cauliflower-like inflorescence tissue from the *sep4t* and *an* mutants. These new data and analyses demonstrate that *sep4t* and *an* cauliflower samples show similar expression profiles (based on PCA). Furthermore, *AN* transcripts are barely detectable in *sep4t*

inflorescence meristem tissue, indicating that *SEP4* activity is required for proper expression of *AN* and that the cauliflower-like phenotype of *sep4t* can be largely attributed to the regulation of *AN* by *SEP4* genes. Interestingly, the *SEP4* genes *J2 and EJ2* are also expressed at low levels in *an* inflorescence tissue, pointing towards regulatory feedback maintenance between *AN* and *SEP4* genes.

We present this new data now in a revised main figure (**Fig. 4I-J**) and new supplementary figure (**Figure S7**):

Figure 4I-J:

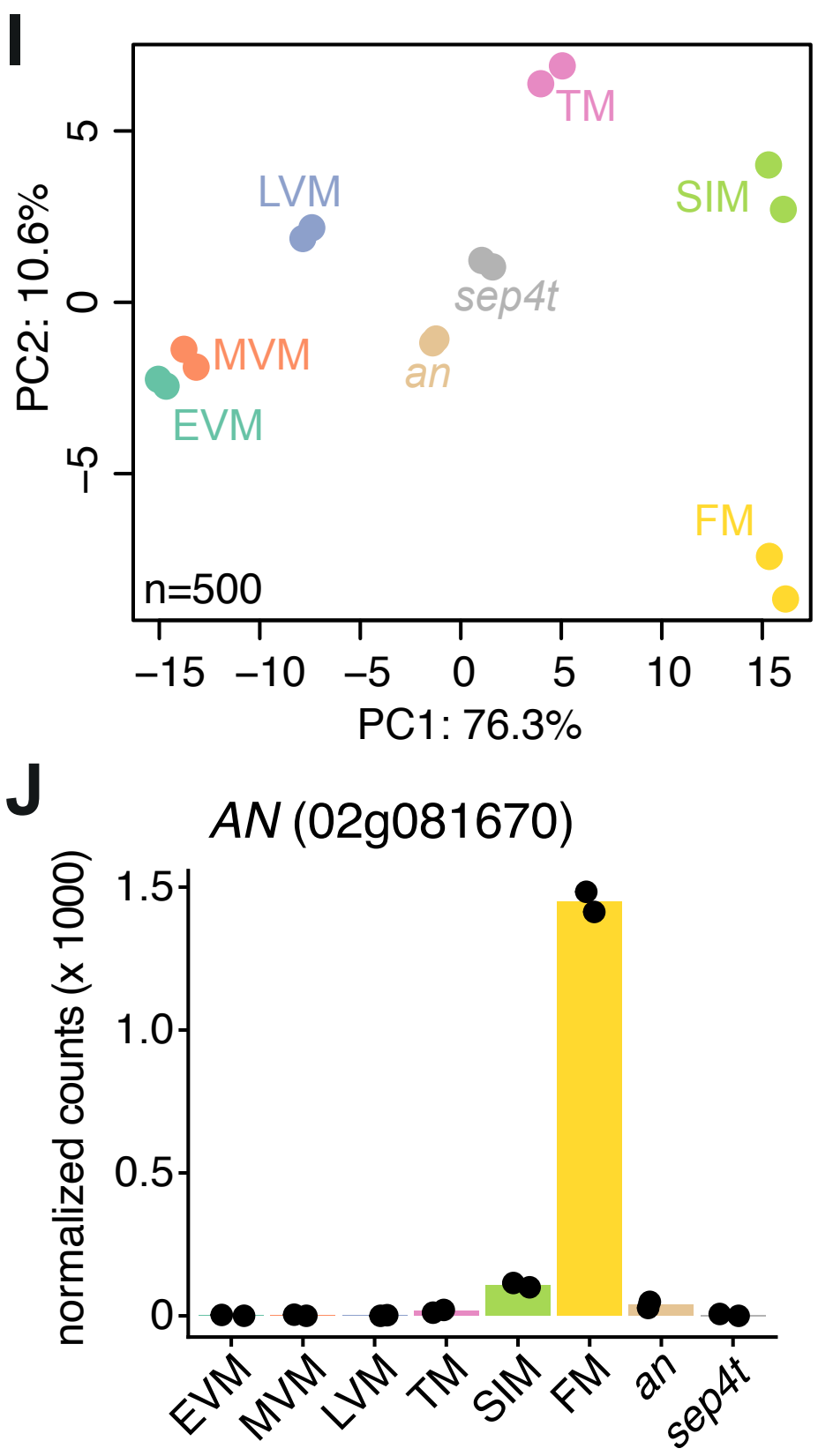

(**I**) PCA of the 500 most differential genes in *sep4t* (*j2 ej2 lin*), and *an* inflorescence tissue and staged WT meristems (EVM, early vegetative; MVM, middle vegetative; LVM, late vegetative; TM, transition; SIM, sympodial inflorescence; FM, floral). (**J**) *AN* expression levels in *an* and *sep4t* inflorescence tissue, and staged WT meristems.

Figure S7:

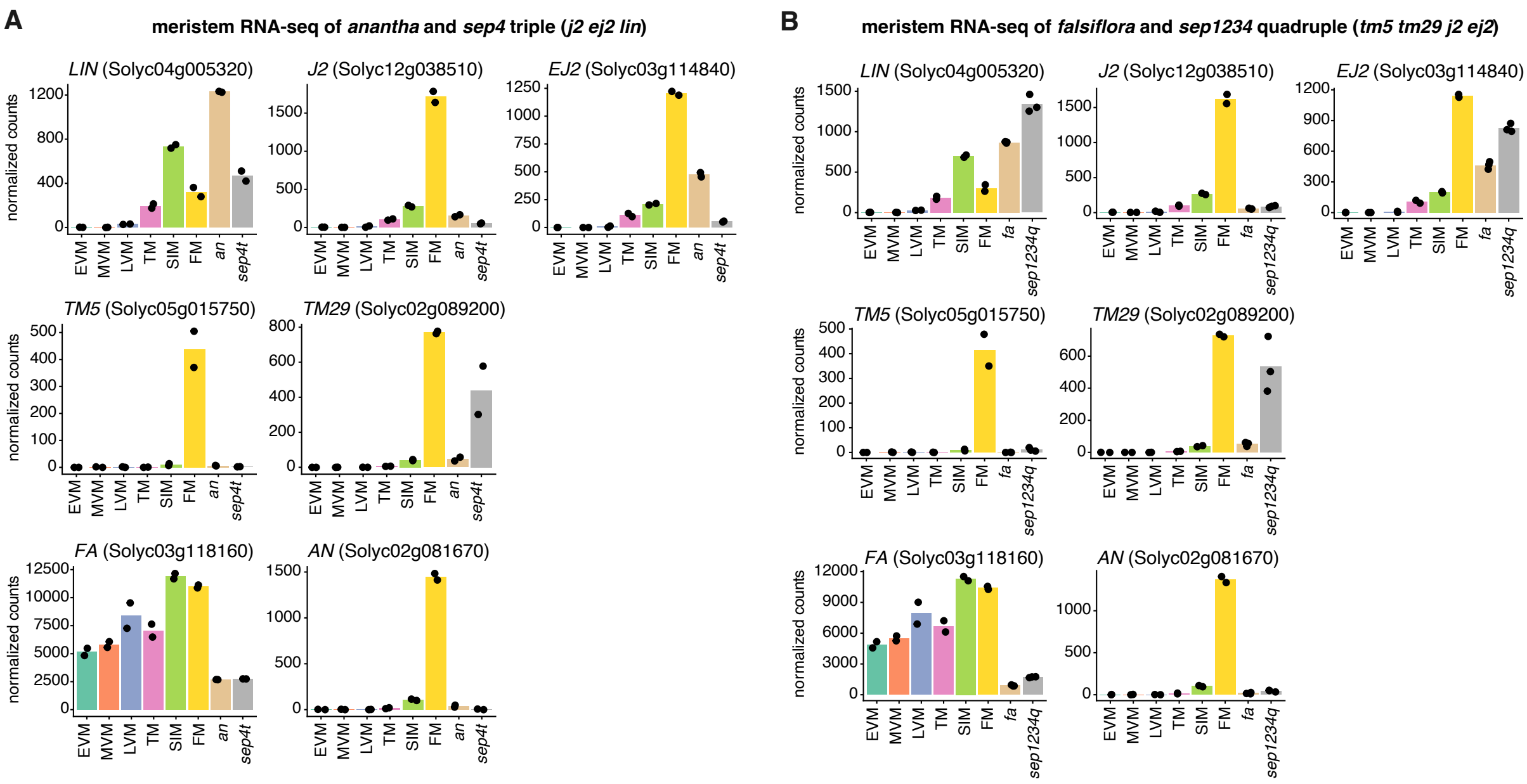

Figure S7: Gene expression levels of *SEP1-4, AN and FA* in WT meristems and *an*, *fa*, *sep4t*, and *sep1234q* mutant inflorescence tissue. (A,B) Expression (normalized read counts) of *SEP1/2* (*TM29*), *SEP3* (*TM5*) and *SEP4* (*J2*, *EJ2*, *LIN*) genes, and *AN/FA* in staged WT meristems compared to *an* and *sep4* triple (*sep4t*; *j2 ej2 lin*) mutant inflorescence tissue (A), or compared to *fa* and *sep1234* quadruple (*sep1234q*; *tm5 tm29 j2 ej2*) mutant inflorescence tissue (B). EVM, early vegetative; MVM, middle vegetative; LVM, late vegetative; TM, transition; SIM, sympodial inflorescence; FM, floral meristem stage of meristem maturation).

In the results on page 7, we now write:

To further test this hypothesis, we profiled gene expression of *an* and *j2 ej2 lin* triple mutant (*sep4t*) inflorescences by RNA-seq. Principal component analyses (PCA) of mutant transcriptomes with staged WT meristem profiles [6] revealed similar transcriptional states of *an* and *sep4t* meristems (**Figure 4I**). In addition, *AN* transcripts were barely detectable in *sep4t* meristems (**Figure 4J**), suggesting that *sep4t* cauliflower inflorescences can be largely attributed to the loss of *AN* expression.

In the results on page 11, we now write:

To further investigate this potential positive maintenance relationship, we examined expression of *FA*, *AN* and *SEP1-4* genes across the *sep4t*, *sep1234q*, *an*, and *fa* RNA-seq datasets (**Figure S7A-B**). In addition to low *AN* transcript levels in *sep4t* (*j2 ej2 lin*) mutants, we found that *J2* and *EJ2* were downregulated in *an*, supporting a positive feedback loop between *SEP4* genes and *AN* (**Figure 6O**). Further, *TM5* and *TM29* were downregulated in both *an* and *fa*, and *AN* and *FA* were also lowly expressed in *sep4t* and *sep1234q*, indicating a second layer of feedback maintenance between *SEP1-3* and *AN/FA* during floral organ specification. Interestingly, *LIN* showed high expression in *an*, *fa*, and *sep1234q* while *TM29* maintained elevated transcript levels in *sep4t* and *sep1234q*, suggesting potential compensatory mechanisms that warrant further investigation (**Figure S7A-B**).

Reviewer 2, Comment 4:

The authors propose that FA acts upstream of SEP4 initially and, later—once AN is induced by SEP4 proteins—FA collaborates with AN and acts upstream of SEP1-3. A positive feedback loop between FA/AN and SEP4 is suggested to maintain the floral state. This section is highly compelling, as it bridges various classes of MADS-box transcription factors with FA/AN, addressing a notable gap in the field. It also contrasts with the situation in petunia, another Solanaceae species that shares many features but exhibits key differences.

Response: We thank the reviewer for this encouraging and insightful comment. We are glad to see that the proposed regulatory relationships between *SEP* genes and the *FA-AN* module are perceived as a meaningful contribution to the field.

As highlighted by the reviewer, comparisons with the Solanaceae species petunia provide an interesting evolutionary perspective since several developmental features are conserved while key differences exist. Functional divergence in petunia has been described in a landmark study by Morel et al., 2019 (PMID: 31591161). In addition to one *SEP3*-clade gene (*FBP2*), petunia encodes two *SEP1/2*-clade genes (*FBP5* and *PMADS12*) and only three *SEP4/FBP9*-clade genes (*FBP4*, *FBP9*, *FBP23*).

As we already write at the end of the discussion, the similarities between tomato and petunia *SEP4/FBP9*-clade genes become clear in the *fbp4 fbp9 fbp23* triple mutant, the genetic equivalent to the *sep4t* (*j2 ej2 lin*) triple mutant, which develops also in petunia highly branched and flowerless inflorescences. However, *fbp4 fbp9 fbp23* inflorescences appear vegetative due to the presence of bracts and are very similar to those of the *alf* mutant, which lacks activity of the petunia *LEAFY* ortholog. This contrasts the situation in tomato where the *sep4t* mutant phenocopies *anantha* (*ufo*) and *sep1234* mutants phenocopy *falsiflora* (*leafy*). Whether these differences are due to species-specific differences in inflorescence morphology, different trajectories during *SEP* functional divergence, and/or *LEAFY/UFO* module function is difficult to address but warrants future comparative cross-species examinations. A notable difference between petunia and tomato is the floral identity function of tomato *SEP4*-clade genes, which has not been reported for the petunia *SEP4/FBP9*-clade genes (*FBP4 FBP9 FBP23*). In tomato, *SEP4* function on floral identity is evident in the *ej2* single mutant (sepal defects) but most notably in *sep1/2/3/4* mutant combinations (*tm5 tm29 j2* and *tm5 tm29 ej2*).

Similarities between tomato and petunia *SEP1/2/3*-clade genes are evident in the *fbp2* (*sep3*) mutant, which is the only petunia *sep* single mutant with obvious floral organ (petal) defects, similar to its equivalent *tm5* in tomato. Furthermore, floral organ defects are dramatically enhanced in the *fbp2 fbp5 pmads12* (*sep1/2/3*) mutant, the equivalent to tomato *tm5 tm29*. Similar to *tm5 tm29*, petunia *fbp2 fbp5 pmads12* develop ovaries

filled with leaf-like organs that represent converted ovules. However, in contrast to the leaf-like stamen of *tm5 tm29*, the stamen of petunia *fbp2 fbp5 pmads12* retain partial stamen identity, suggesting that other genes can compensate for the loss of *SEP1/2/3* function in petunia.

Given the already substantial length of the manuscript (see also reviewer 1 comment 1), we refrained from an in-depth discussion of these species-specific differences, which we believe would be an excellent topic for a review article. However, to better highlight at least some *SEP1/2/3* differences between tomato and petunia, we now write in the results on page 9:

In addition, *tm5 tm29* flowers gave rise to parthenocarpic (seedless) fruits that contained leaf-like organs instead of seeds (**Figure 5I** and **Figure S4H-I**), similar to petunia *sep123* (*fbp2 fbp5 pmads12*) mutants, which develop ovaries filled ovules converted to leaf-like organs. In contrast to tomato *tm5 tm29*, petunia *fbp2 fbp5 pmads12* flowers retain partial stamen identity [48].

Reviewer 2, Comment 5:

What I find most lacking in this work is an analysis using in situ hybridization to spatially validate the main inferred regulatory interactions: the three SEP4 genes in wild-type vs. fa backgrounds, FA and AN in the triple sep4 mutant, and fa and the three sep4 genes in the an background. Some of these data may already exist in the literature and could be incorporated.

Response: We appreciate the reviewer's critical comment, which highlights an important limitation of this manuscript. We agree that spatial expression analyses of *FA*, *AN*, and *SEP* genes would be very insightful to better understand the regulatory interaction between *FA/AN* and *SEP* genes. We now incorporate published RNA *in situ* hybridization experiments in the manuscript and discuss expression domains for the *SEP1-3* genes *TM5* and *TM29*, the *SEP4* gene *J2*, as well as *FA* and *AN*, which have been shown to be expressed in the dome of floral meristem in the WT.

In the results on page 8 we now write:

Previous studies had demonstrated by RNA in-situ hybridization that *TM5* and *TM29* are expressed in floral meristems at the onset of organogenesis and that individual knock-down by antisense technology transforms carpels, the female reproductive floral organs that give rise to the tomato fruit, into ectopic inflorescences [45,46].

In the discussion on page 14 we now write:

Thus, transcriptional feedback regulation between *SEP1/2/3/4* paralogs and *AN/FA* (**Figure 6O**) ensures a critical *SEP* dosage to maintain *FA* and *AN* expression after the floral transition for accurate floral meristem patterning and floral organ differentiation. This model warrants

further investigation using spatially resolved approaches to delineate feedback maintenance between *SEP* and *AN/FA* genes. While the spatiotemporal expression domains of *TM5* [45], *TM29* [46], *J2/SLMBP21* [58], *AN* [18], and *FA* [15] in floral meristems have been characterized by in situ hybridization experiments at tissue level individually, examining how these patterns are modified in *sep* and *an/fa* mutant backgrounds at cellular resolution will provide additional mechanistic insights.

We also agree that it is important to understand whether and how *FA-AN* expression is affected in the *sep4* triple mutant, and *SEP1-4* expression is affected in the *fa* and *an* mutant. The reviewer points out a relevant set of *in-situ* experiments, i.e. the three *SEP4* genes in WT, *fa*, and *an*, and *AN* and *FA* in the WT and *sep4t* mutant, which presents an extensive set of additional experiments that we think fall beyond the scope of this study. However, to at least partially address this comment, we present now an additional RNA-seq dataset for *sep4t* (*j2 ej2 lin*) and *an* mutant inflorescence meristem tissue, which helps address some of the questions raised. We present this new data in the response to Reviewer2's comment 2. In brief, we found that *AN* is barely detectable in *sep4t*, further supporting that *J2*, *EJ2* and *LIN* redundantly activate *AN*. Conversely, *J2* and *EJ2* transcript levels are low in *an* mutants, suggesting that *AN* activity also promotes *J2* and *EJ2* expression, consistent with positive feedback. We present this new data in a revised main figure (**Fig. 4I-J**) and a new supplementary figure (**Figure S7**).

Reviewer 2, Comment 6:

Finally, a critical question remains: Why are both SEP4 and FA/AN required to initiate flower meristem development and SEP1-3 induction? In situ hybridization experiments (e.g., SEP1-3 in fa, an, and triple sep4 backgrounds) could be performed or at least discussed. Given what is known in other plants—and the observation (line 386) that no J2 binding sites are found in SEP1-3—I would not be surprised if FA/AN or FA dimers serve as the primary upstream regulators.

Response: We thank the reviewer for this thoughtful comment. We agree that it is a good question "why" both *SEP4* and *FA/AN* module should be required to induced *SEP1-3* expression and floral organ initiation. Placing the *FA/AN* module as an upstream or intermediate factor is an attractive model.

As described in the response to Reviewer 2's comment 3, we conducted additional RNA-seq analyses on *sep4t* and *an*, and analyzed the expression patterns of *SEP1-4*, *FA*, and *AN* across the *sep4t*, *sep1234t*, *an*, and *fa* mutant datasets. Based on this study, published datasets, and the reviewer's comment, we revised our model for the regulatory feedback between *SEP1-4* genes and *AN/FA*. In brief, we propose a sequential, positive feedback between *SEP4* genes and *SEP1-3* genes with *FA/AN* as intermediate factor that bridges subsequent stages of floral meristem development:

During the acquisition of floral identity, *SEP4* genes induce *AN* expression In support of this regulatory interaction, *AN* expression is downregulated in *j2 ej2* mutants (this study), and J2 binds upstream of *AN* (Wang et al., 2023, PMID: 36881857) to a functional enhancer element (Lanctot et al., 2025, PMID: 39964724), strongly suggesting that J2 directly activates *AN*. Furthermore, *AN* transcripts are barely detectable in *sep4* triple mutants (*j2 ej2 lin*) (this study), suggesting that *J2*, *EJ2* and *LIN* redundantly promote *AN* expression. Conversely, *J2* and *EJ2* transcript levels are very low in *an* mutants (this study), suggesting that *AN* promotes their expression, consistent with a positive feedback loop. Whether this regulation is direct or indirect we cannot determine due to the lack of FA-FA and FA-AN ChIP-seq data, although we identified predicted *LFY* binding sites upstream of *J2* (unpublished data).

During floral meristem patterning and organ development, *AN/FA* promote *SEP1-3* expression. In support of this regulatory interaction, *SEP1-3* (*TM5* and *TM29*) expression is reduced in *falsiflora* meristems (this study), which is consistent with the situation in Arabidopsis, where *LFY* directly activates *SEP3* transcription (Winter et al., 2011, PMID: 21497757). Conversely, *SEP1-3* activity is required to maintain *FA* expression during floral organ development and floral meristem patterning, as *sep1234* quadruple mutants (*j2 ej2 tm5 tm29*) phenocopy *falsiflora* null mutants by developing highly branched inflorescences that fail to develop flowers (this study). Partial loss of *FA* activity caused by CRISPR-induced chimerism (this study) or CRISPR-induced promoter variation (Quevedo-Colmena et al., 2025, Curr Plant Biol) leads to floral organ defects like those observed in *sep123* mutants (*tm5 tm29*). In addition, *FA* transcript levels are strongly reduced in *sep1234q* mutants (this study), and J2 binding upstream of *FA* (Wang et al., 2023, PMID: 36881857) suggests that *SEP* genes redundantly maintain *FA* expression after floral identity is established.

Together, these observations support a model in which sequential regulatory interactions between *SEP4* genes, *FA/AN*, and *SEP1-3* genes coordinate the transition to floral identity and its maintenance during floral organ development. We describe this revised model now in with schematics in **Fig. 6O** but acknowledge that not all regulatory relationships are mechanistically resolved.

Minor comments:

Reviewer 2, Comment 7:

* A summary schematic (GRN) highlighting the newly identified regulatory interactions—and the confidence level in each—would be a valuable addition to the final figure.

Response: We thank the reviewer for this helpful suggestion. We agree that a summary schematic would enhance the clarity of the manuscript. In this revised version, we have added a new panel to the final figure (**Fig. 6O**), in which we integrate expression patterns and functional interactions between *SEP* and *FA/AN* genes that we identified by genetic, transcriptomic, and published ChIP-seq data. Based on this data, we also distinguish the level of confidence (data support) for each of the regulatory interactions. Together with a second model (**Fig. S4L)**, requested by Reviewer 1, which illustrates the level of redundancy of *SEP* genes during floral organ specification, we believe that we improved the clarity of our findings and interpretations.

Reviewer 2, Comment 8:

* The excellent reviews by Claire Perilleux's team could be cited in the introduction and discussion.

Response: We thank the reviewer for this helpful suggestion. We have now included two very relevant reviews from the Périlleux lab (Périlleux et al., 2019, Trends Plant Sci and Linden et al., 2026, Trends Plant Sci) in the Introduction and Discussion, where they provide important context for the interpretation of our findings.

On page 2 in the introduction, we now write:

The timely control of meristem maturation towards reproductive fate ensures inflorescence development [5].

(Ref. 5: Périlleux C, Bouché F, Randoux M, Orman-Ligeza B. Turning Meristems into Fortresses. *Trends Plant Sci*. 2019;24(5):431-442. doi:10.1016/j.tplants.2019.02.004)

On page 13 in the discussion, we now write:

Together, these findings show that *AP1/FUL*-genes are involved in both guiding meristem transitions and specifying floral organ identity, paralleling the roles of *FA* and *SEP* genes described in this study. This dual function supports a model in which the floral transition and floral organogenesis represent a developmental continuum rather than distinct phases [57].

And on page 14 in the discussion, we now write:

UFO forms a ring-like expression domain in vegetative and inflorescence meristems, which has been proposed to establish a pattern that in combination with local activation of *LFY* facilitates floral meristem initiation and bract suppression [57].

(Ref. 57: Linden J, Gaydardzhiev K, Bouché F, Périlleux C. Flowering at the phytomer scale. *Trends Plant Sci*. Published online March 27, 2026. doi:10.1016/j.tplants.2026.01.002)

Reviewer 2, Comment 9:

* The recent work by Jiang et al. (2025, New Phytologist) should also be cited, particularly as the MADS-box binding in FA might involve regulation by AP1/FUL genes.

Response: We thank the reviewer for this helpful suggestion. We now cite the work by Jiang et al., 2025 (PMID: 40827093) and also tried to integrated their findings with our results in a new paragraph in the discussion. We describe this integration in detail in the response to Reviewer 2, comment 2.

Reviewer #3:

Reviewer 3, comment 1

This manuscript presents some beautiful mutant phenotypes of the three SEP4 paralogs in tomato (including single mutants and various combinatorial mutants) as well as paralogs of the three SEP clades (TM5/TM29/SEP4). Additional transcriptomic and functional data suggest that the SEP proteins directly regulate AN/UFO and FA/LEAFY expression. The latter point is significant because UFO regulation is still poorly understood, despite its critical importance in inflorescence/flower development.

Response: We appreciate the reviewer's positive assessment of our manuscript and their recognition of our functional genetic and transcriptomic analyses. We are particularly pleased that the proposed regulation of *AN/UFO* and *FA/LEAFY* by SEP proteins is recognized as a significant aspect of this study. In the revised version of the manuscript we present now additional expression analyses (see revised **Fig. 4I-J** and new **Fig. S7**) that provide further evidence for the regulatory feedback link between *SEP* genes and *AN/FA*, and better explain these findings in a schematic model (**Fig. 6O**). Please also see our response to reviewer 2's comments 3, 5, 6.

Reviewer 3, comment 2

Although I find the data valuable and the figures appealing, the conceptual framework of the paper seems odd: isn't it already well established that genetic synergy usually arises from functional redundancy? This study doesn't really contribute anything new in that regard. I would encourage the authors to reframe the study, perhaps just simply "dissecting the genetic redundancy of the SEP genes and their target genes".

Response: We appreciate the reviewer's critical comment regarding the conceptual focus of the study and manuscript. We agree that already previous works provided empirical support for functional redundancy as source of genetic synergy, most notably in yeast (reviewed by Boone et al., 2007, PMID: 17510664) but also in plants (reviewed by Pérez-Pérez et al., 2009, PMID: 19665253, ref. 2). However, we believe that our genetic and transcriptomic analyses across multiple mutant combinations of redundant *SEP* genes provide rare insights into the distinct modes of non-additive interactions, including synergy and less-than-additive effects, and provide a better understanding of the molecular consequences of functional redundancy and how they express in synergistic phenotypes. As also highlighted by Reviewer 2 (comment 2), our study provides a rare analysis of how different modes of genetic interactions manifest at both the transcriptional and phenotypic level. We believe that this work contributes to a better understanding of how synergistic interactions shape not only floral development

in tomato but also more broadly developmental processes in other organisms. However, we realize that we may have overgeneralized the novelty of our statements in several instances in the manuscript. In response to the reviewer's suggestion, we have thus revised the title and abstract, and toned down several generalized statements throughout the manuscript to better point out the novel perspective or the study.

We changed the title to make it less general and more specific to the study of tomato MADS-box genes:

Incomplete functional divergence drives MADS-box gene synergy during floral development in tomato

We modified several sentences in the abstract to make our findings specific to the *SEP*-clade genes and highlight the novel regulatory link between *SEP* genes and the *FA/AN* module:

Genetic synergy arises from interactions between functionally related genes that control complex traits in plants and animals. Synergy occurs when the combined effect of multiple genes exceeds the additive contribution from each individual gene. However, genetic mechanisms that drive and maintain synergy remain underexplored. Here, we investigated synergistic interactions among *SEPALLATA* (*SEP*) MADS-box genes during floral development in tomato. We discovered that *SEP* gene synergy emerges from duplicated genes that partitioned functions to regulate inflorescence architecture and floral organ identity. Moreover, synergistic interactions are reflected in non-additive expression changes that coordinate successive developmental stages. Finally, we demonstrate that *SEP* gene synergy occurred due to residual redundancy on the dose-sensitive *FALSIFLORA/ANANTHA* (*FA/AN*) module guiding floral identity. These results indicate that *SEP* gene synergy emerged as a consequence of incomplete functional divergence under gene dosage constraints. Our work provides insights into mechanisms through which gene families diverge to produce the substrate for biological innovations during evolution.

In the discussion on page 12, we changed the headline and first sentence to:

***SEP* gene synergy emerged from redundancy on the conserved *FA/AN* module.**

In this study, we dissected synergistic interactions between closely related *SEP4*-clade MADS-box genes in the model crop tomato.

In the discussion on page 13, we now write:

Feedback regulation between *SEP* paralogs calibrates their gene dosage.

In the discussion on page 14, we now write:

Gene dosage effects maintain *SEP* redundancy but constrain functional divergence.

A couple of minor comments:

Reviewer 3, comment 3

line 27-29: Again, functional redundancy is the most straightforward explanation of genetic synergy. This sentence is trying hard to package the results of this study in a way that sounds different, but in essence it's still the same as "functional redundancy".

Response: We appreciate the reviewer's critical comment regarding the interpretation of genetic synergy. As mentioned in our response to your second comment, we agree that functional redundancy provides a straightforward explanation for synergistic interactions. Our intention was to emphasize the finding that functional divergence of *SEP* paralogs is constrained by their redundant function on the dose-sensitive *FA/AN* regulatory module. We now revised the sentence in the abstract to better highlight this interpretation.

In line 27 of the abstract, we now write:_

Finally, we demonstrate that *SEP* gene synergy occurred due to residual redundancy on the dose-sensitive *FALSIFLORA/ANANTHA* (*FA/AN*) module guiding floral identity. These results indicate that *SEP* gene synergy emerged as a consequence of incomplete functional divergence under gene dosage constraints.

Reviewer 1, comment 4

line 359-362: The strength of the synergistic effects probably depends more on the degree of expression overlap between the paralogs instead of evolutionary distance.

Response: We appreciate the reviewer's thoughtful comment. We agree that one factor influencing the strength of synergistic effects is the degree of expression overlap between paralogs, which often declines as paralogs diverge over evolutionary time. Another factor that can influences synergy between paralogs is variation in protein coding sequence. We now cite an excellent recent review by Cisneros et al., 2026 (PMID: 41699381, ref. 53) about the evolutionary causes and consequences of gene duplication to better acknowledge the molecular mechanisms that are known to modify paralog redundancy over evolutionary time.

We now write in the discussion on page 12:

Here, we found that *SEP4* synergy on gene expression programs underlying inflorescence branching is more pronounced between the direct paralogs *J2* and *EJ2* than with the distant

paralog *LIN*. This pattern is consistent with paralog redundancy generally declining over evolutionary time, likely driven by divergence in coding sequences and gene expression patterns[53].
