# Supplementary material for: Incomplete functional divergence drives MADS-box gene synergy during floral development in tomato
Source: PLoS Biol. 2026 Aug 18;24(8):e3003946. doi: 10.1371/journal.pbio.3003946 (PMC13506103; doi:10.1371/journal.pbio.3003946)
Supplement: Supplementary file 2 [file pbio.3003946.s028.docx]

**Response to Editorial and reviewer comments**

We thank the three reviewers and the editors for their time and effort in reviewing our manuscript. We are pleased to hear that you are in principle accepting our manuscript for publication. Please find below our detailed responses to all comments.

**Editorial office - comments:**

a) Please address my Data Policy requests below; specifically, we need you to supply the numerical values underlying Fig. 2C, H, I, J; Fig. 3A-D, G, H; Fig. 4A, B, D, E, G, I, J; Fig. 5A; Fig. 6J, L, M, N; Fig. S1A; Fig. S2A, B, C, D; Fig. S4A, L; Fig. S5C; Fig. S6A-C; Fig. S7A, B either as a supplementary data file or as a permanent DOI’d deposition (such as Zenodo).

**Response:** We a new supplementary data file (Supplementary Data 1) with all numerical values that underlie the indicated figures.

The panels Fig. 4A and Fig. S4L are not based on numerical values but are schematic representation or models as indicated in the respective figure legend:

**Figure legend of Figure 4: (A)** Schematic representation of additive and non-additive gene expression categories in double mutants when compared with single mutants.

**Figure legend of Figure S4:** (**L**) Model depicting the level of *SEP* gene redundancy during floral organ development in tomato.

b) Please clearly cite the location of the data in all relevant main and supplementary figure legends, for example: “The data underlying this figure can be found at [doi.org/10.xxx/zenodo.xxxxx](http://doi.org/10.xxx/zenodo.xxxxx).” We understand that this may appear repetitive, but it helps make the figures and their legends more self contained.

**Response:** We now cite the Supplementary Data 1 file in all relevant main and supplementary figure legends, for example in the legend of figure 2 we write “The data underlying this figure can be found in Supplementary Data 1”.

c) Please make the raw data deposited on SRA publicly available at this stage.

**Response:** We released the BioProject PRJNA1348489 on July 13, 2026.

d) Please make any custom code available (if any), either as a supplementary file or as part of your data deposition.

**Response:** We made all relevant custom code available on GitHub and Zenodo and cite the repositories in the Data Availability Statement.

As you address these items, please take this last chance to review your reference list to ensure that it is complete and correct. If you have cited papers that have been retracted, please include the rationale for doing so in the manuscript text, or remove these references and replace them with relevant current references. Any changes to the reference list should be mentioned in the cover letter that accompanies your revised manuscript.

**Response:** We reviewed the reference list to ensure that it is complete and correct. As suggested by Reviewer 3, we added one reference:

5.	Périlleux, C. & Huerga-Fernández, S. Reflections on the Triptych of Meristems That Build Flowering Branches in Tomato. *Front. Plant Sci.* **13**, (2022).

**Editorial office – formatting changes:**

1) We do not publish any copyright or trademark symbols that usually accompany proprietary names, eg (R), (C), or TM (e.g. next to drug or reagent names). Please remove all instances of trademark/copyright symbols throughout the text, including Arcturus™ ,PicoPure™ on line no 594

**Response:** We removed all “TM” symbol from the manuscript.

2) We have noticed that you have uploaded "Supplementary Data" file as supporting information but you have not included it in the list of legends. Please add a citation of this SI file into the list of legends of your supporting information files.

**Response:** We included citation for all Supplementary Data in the list of legends.

3) In your Data Availability Statement, please ensure you have provided all necessary access information in complete sentences. Access information should include the name of any data repositories used as well as digital object identifiers (DOIs), accession numbers or codes, or other persistent identifiers for all relevant data. If all data has been provided within your submission files, please use the statement "All relevant data is available within the manuscript and Supporting Information files." The Data Availability Statement will appear on the published version of your manuscript.

**Response:** We confirm that the Data Availability Statement provides all necessary information.

4) Please refer to the following article for PLOS Biology's best-practice recommendations for data presentation: <https://journals.plos.org/plosbiology/article?id=10.1371/journal.pbio.1002128>.
Guidelines for statistical reporting, if relevant, may be found here: <https://journals.plos.org/plosbiology/s/best-practices-in-research-reporting#loc-statistical-reporting>.

**Response:** Thank you for the guidance.

**Reviewer #1:**

The authors have done a thorough job addressing each of the reviewers' comments, both through the additional of new data/figures/text or arguing their case. I'm happy with the changes and believe this paper will make a strong contribution to our understanding of functional divergence, MADS-box gene evolution, and mechanisms of plant development.

**Response:** Thank you for this positive comment. We really appreciated your detailed and thoughtful comments on the first version, which helped us to significantly improve the clarity of the manuscript. We are glad to see tha you are satisfied with the revised version.

**Reviewer #2:**

Reviewer #2: I have read with interest the new version of this manuscript as well as the responses to the referees. I already had a very good opinion of the first version, and I asked for a few things without claiming any of them would be absolutely required.

I am very satisfied with the new version. I appreciate that the title was slightly amended. I think the addition of the RNA-seq in sep4t and an add a lot in terms of regulatory network and largely compensate for the lack of in situ hybridization. Moreover, I think the little summary scheme presented in Figure 6 is very useful for the readers.

Overall, I think this work represents a great contribution to the field of inflorescence and flower development in tomato and in angiosperms in general.

Little detail: I had in mind the tomato review by Perilleux et al. (<https://doi.org/10.3389/fpls.2022.798502>), even if the Arabidopsis reviews are also of high quality.

**Response:** Thank you for your positive comment, we are glad to see that you are satified with the revised version. We would also like to thank you for your critical comments on the first version, which helped us to improve the manuscript.

**Reviewer #3:**

Reviewer #3: The authors did a great job in revising the manuscript and addressed my comments satisfactorily.

Response: Thank you for this positive comment, we would also like to thank you for your critical comments on the first version.
