# Supplementary material for: Incomplete functional divergence drives MADS-box gene synergy during floral development in tomato
Source: PLoS Biol. 2026 Aug 18;24(8):e3003946. doi: 10.1371/journal.pbio.3003946 (PMC13506103; doi:10.1371/journal.pbio.3003946)
Supplement: Peer review history

# Editor report: Decision Letter 0

**DOI:** 10.1371/journal.pbio.3003946.r001

**Authors:** Ankiit Ahluwalia

9 Jan 2026

Dear Dr Soyk,

Thank you for submitting your manuscript entitled "Incomplete functional divergence drives genetic synergy during floral development in tomato" for consideration as a Research Article by PLOS Biology.

Your manuscript has now been evaluated by the PLOS Biology editorial staff [as well as by an academic editor with relevant expertise] and I am writing to let you know that we would like to send your submission out for external peer review.

However, before we can send your manuscript to reviewers, we need you to complete your submission by providing the metadata that is required for full assessment. To this end, please login to Editorial Manager where you will find the paper in the 'Submissions Needing Revisions' folder on your homepage. Please click 'Revise Submission' from the Action Links and complete all additional questions in the submission questionnaire.

Once your full submission is complete, your paper will undergo a series of checks in preparation for peer review. After your manuscript has passed the checks it will be sent out for review. To provide the metadata for your submission, please Login to Editorial Manager (https://www.editorialmanager.com/pbiology) within two working days, i.e. by Jan 13 2026 11:59PM.

If your manuscript has been previously peer-reviewed at another journal, PLOS Biology is willing to work with those reviews in order to avoid re-starting the process. Submission of the previous reviews is entirely optional and our ability to use them effectively will depend on the willingness of the previous journal to confirm the content of the reports and share the reviewer identities. Please note that we reserve the right to invite additional reviewers if we consider that additional/independent reviewers are needed, although we aim to avoid this as far as possible. In our experience, working with previous reviews does save time.

If you would like us to consider previous reviewer reports, please edit your cover letter to let us know and include the name of the journal where the work was previously considered and the manuscript ID it was given. In addition, please upload a response to the reviews as a 'Prior Peer Review' file type, which should include the reports in full and a point-by-point reply detailing how you have or plan to address the reviewers' concerns.

During the process of completing your manuscript submission, you will be invited to opt-in to posting your pre-review manuscript as a bioRxiv preprint. Visit http://journals.plos.org/plosbiology/s/preprints for full details. If you consent to posting your current manuscript as a preprint, please upload a single Preprint PDF.

Feel free to email us at plosbiology@plos.org if you have any queries relating to your submission.

Kind regards,

Ankiit

Ankiit Ahluwalia,

PLOS Biology

aahluwalia@plos.org

# Peer review: Decision Letter 1

**DOI:** 10.1371/journal.pbio.3003946.r002

**Authors:** Ankiit Ahluwalia

26 Feb 2026

Dear Dr Soyk,

Thank you for your patience while your manuscript "Incomplete functional divergence drives genetic synergy during floral development in tomato" was peer-reviewed at PLOS Biology. It has now been evaluated by the PLOS Biology editors, an Academic Editor with relevant expertise, and by three independent reviewers.

In light of the reviews, which you will find at the end of this email, we would like to invite you to revise the work to thoroughly address the reviewers' reports.

You will see that all three reviewers find the study interesting and worth pursuing, but they also raise several issues that would need to be addressed before we can consider the manuscript for publication. Reviewer #1 suggests adding a final overview figure illustrating the different levels of redundancy among the SEP genes, along with a few minor comments for clarity. Reviewer #2 thinks that performing in situ hybridization would spatially validate the main regulatory interactions, or at least existing data available in the literature could be discussed. In addition, this reviewer proposes adding a summary schematic (a GRN) highlighting the newly identified regulatory interactions to complement the final figure. Reviewer #3 recommends reframing the study, perhaps simply as “dissecting the genetic redundancy of the SEP genes and their target genes.”

After discussing these comments with the Academic Editor and the rest of the team, we have decided to invite a revision that should address all the points raised by the reviewers. We agree with Reviewer #2 that additional in situ experiments would strengthen the paper, but if there are some already available in the literature, you may add them.

Given the extent of revision needed, we cannot make a decision about publication until we have seen the revised manuscript and your response to the reviewers' comments. Your revised manuscript is likely to be sent for further evaluation by all or a subset of the reviewers.

In addition to these revisions, you will need to complete some formatting changes, which you will receive in a follow up email. A member of our team will be in touch with a set of requests shortly.

We expect to receive your revised manuscript within 3 months. Please email us (plosbiology@plos.org) if you have any questions or concerns, or would like to request an extension.

At this stage, your manuscript remains formally under active consideration at our journal; please notify us by email if you do not intend to submit a revision so that we may withdraw it.

**IMPORTANT - SUBMITTING YOUR REVISION**

Your revisions should address the specific points made by each reviewer. Please submit the following files along with your revised manuscript:

1. A 'Response to Reviewers' file - this should detail your responses to the editorial requests, present a point-by-point response to all of the reviewers' comments, and indicate the changes made to the manuscript.

*NOTE: In your point-by-point response to the reviewers, please provide the full context of each review. Do not selectively quote paragraphs or sentences to reply to. The entire set of reviewer comments should be present in full and each specific point should be responded to individually, point by point.

You should also cite any additional relevant literature that has been published since the original submission and mention any additional citations in your response.

2. In addition to a clean copy of the manuscript, please also upload a 'track-changes' version of your manuscript that specifies the edits made. This should be uploaded as a "Revised Article with Changes Highlighted" file type.

*Re-submission Checklist*

When you are ready to resubmit your revised manuscript, please refer to this re-submission checklist: https://plos.io/Biology_Checklist

To submit a revised version of your manuscript, please go to https://www.editorialmanager.com/pbiology/ and log in as an Author. Click the link labelled 'Submissions Needing Revision' where you will find your submission record.

Please make sure to read the following important policies and guidelines while preparing your revision:

*Published Peer Review*

Please note while forming your response, if your article is accepted, you may have the opportunity to make the peer review history publicly available. The record will include editor decision letters (with reviews) and your responses to reviewer comments. If eligible, we will contact you to opt in or out. Please see here for more details:

https://blogs.plos.org/plos/2019/05/plos-journals-now-open-for-published-peer-review/

*PLOS Data Policy*

Please note that as a condition of publication PLOS' data policy (http://journals.plos.org/plosbiology/s/data-availability) requires that you make available all data used to draw the conclusions arrived at in your manuscript. If you have not already done so, you must include any data used in your manuscript either in appropriate repositories, within the body of the manuscript, or as supporting information (N.B. this includes any numerical values that were used to generate graphs, histograms etc.). For an example see here: http://www.plosbiology.org/article/info%3Adoi%2F10.1371%2Fjournal.pbio.1001908#s5

*Blot and Gel Data Policy*

We require the original, uncropped and minimally adjusted images supporting all blot and gel results reported in an article's figures or Supporting Information files. We will require these files before a manuscript can be accepted so please prepare them now, if you have not already uploaded them. Please carefully read our guidelines for how to prepare and upload this data: https://journals.plos.org/plosbiology/s/figures#loc-blot-and-gel-reporting-requirements

*Protocols deposition*

To enhance the reproducibility of your results, we recommend that if applicable you deposit your laboratory protocols in protocols.io, where a protocol can be assigned its own identifier (DOI) such that it can be cited independently in the future. Additionally, PLOS ONE offers an option for publishing peer-reviewed Lab Protocol articles, which describe protocols hosted on protocols.io. Read more information on sharing protocols at https://plos.org/protocols?utm_medium=editorial-email&utm_source=authorletters&utm_campaign=protocols

Thank you again for your submission to our journal. We hope that our editorial process has been constructive thus far, and we welcome your feedback at any time. Please don't hesitate to contact us if you have any questions or comments.

Sincerely,

Ankiit

Ankiit Ahluwalia,

PLOS Biology

aahluwalia@plos.org

------------------------------------

REVIEWS:

Reviewer #1: In their manuscript entitled "Incomplete functional divergence drives genetic synergy during floral development in tomato", Gaarslev et al. aim to determine the reason for non-additive inflorescence phenotypes for single, double, and triple sep4-like mutants in tomato. They are specifically interested in the developmental genetic basis of differences in "synergistic" interactions between paralogous pairs, whereby loss-of-function mutations at two or more loci have a more dramatic phenotypic effect that mutations in the single mutants combined. To do this, the authors characterize inflorescence phenotypes and compare differentially expressed gene expression in falsiflora (fa) and sep1/2/3/4 mutant combinations. Results showed that j2:ej2 double mutants had more synergistic inflorescence phenotypes than lin:j2 or lin:ej2 double mutants, and that SEP1/2/3 are partially redundant with SEP4 genes in conferring floral organ identity/determinacy. The authors also identified ANANTHA (AN)and possibly FA as direct targets of SEP4-like proteins that is responsive to SEP4-like dosage effects.

The manuscript is well written and logically laid out. Despite the Results being rather long, they were easy to read. The authors clearly conducted a lot of experimental work for this manuscript, and I found the analyses to be appropriate for the study. I know there are already quite a few main figures. However, given all this work, I wonder whether the authors would consider adding a final overview figure, illustrating the different levels of redundancy for the SEP genes. There was a nice one in Fig. 3a in Rodriguez-Leal et al. (2019) Nature Genetics 51: 786-792 that was on genetic compensation in tomato. Other than that, my comments are minor and outlined below.

Line 51: should this be "transitional meristem"? I haven't encountered "transition meristem" before but maybe this is a tomato term.

Line 60: should this say "In tomato mutants"? Or is this referring to natural variation in tomato?

Line 66: I think this should say Arabidopsis thaliana at first mention.

Line 90: perhaps reword to "…expanded to include genes with E-class function,…)?

Line 102: given what you've already said, perhaps reword to "In addition to J2, EJ2 and LIN, the tomato genome encodes a fourth SEP4 paralog RIPENING INHIBITOR (RIN).

Line 128: do the j2 ej2 double mutants that produce flowers also show the single mutant phenotypes of loss of fruit abscission and elongated sepals? What about the other mutants?

Lin 135: I'd suggest saying "between J2 and EJ2" rather than "J2/EJ2" since the latter usually denoted two genes as a unit.

Line 191: Rather than "DEGs unlikely explain", I suggest "DEGs are unlikely to explain".

Line 239: Maybe it is a journal requirement, but usually family and subfamily names are not in italics.

Line 259: should be "floral organ specification" not "organs".

Line 285: what do you mean by "SEP4 is partially lost"? Do you mean SEP4 function?

Line 342: "as a reference" rather than "as reference"?

Line 361: I think I'd say "indicating that synergistic effects declined with evolutionary distance" or "indicating that synergistic effects may generally decline…". The way it is worded now suggests a statement of fact about synergy, which I don't think the authors can declare with a single example.

Line 367: why "predetermined" and not just "determined"? To have made a decision does not necessarily mean steps have been taken toward the decided goal.

Line 419: I'm curious as to what evidence is available to say that SEP3 is the ancestral paralogue since paralogues are by their very nature equidistant to each other in a phylogenetic sense. Is this based on synteny or does the SEP3 sequence better resemble what we think the ancestral (i.e. pre-duplication) SEP sequence looked like?

Line 450-456 (and elsewhere): numbers below 11 are usually spelled out in full.

Line 470-473: perhaps remove one of the "Briefly"s.

Reviewer #2: Through phenotypic characterization of multiple SEP4 mutants (affecting the three MADS-box genes from the SEP4 family: LIN, J2, and EJ2), combined with gene expression analyses, this study establishes the relative importance of the various SEP4 genes in tomato flower development. It then focuses on several known SEP4 targets—key regulators—to deepen our understanding of the gene regulatory network (GRN) controlling flower development (involving AN, FA, and SEP1-3).

This manuscript presents a substantial body of high-quality data and analyses, with visually compelling figures. While some results are novel and exciting, others are more descriptive and confirmatory. Overall, the work represents a significant contribution to elucidating how flowers develop in tomato.

The analysis of gene expression in the three double mutants affected in J2, EJ2, and LIN is particularly interesting. It not only identifies differentially expressed genes (DEGs) but also explores the nature of their regulation. A less-than-additive effect suggests that the first SEP mutation severely impairs the activity of the protein complex, rendering a second mutation less impactful—implying that the complex likely contains two SEP4 members. Conversely, strong synergy indicates redundancy, where one mutated SEP4 member can be almost fully compensated by another (and thus a distinct complex). To my knowledge, this is a rare and highly informative analysis. However, the study does not attempt to link these observations to the actual composition of heterotetrameric complexes or their regulatory signatures (e.g., CArG boxes) in the DEGs. It would be valuable to speculate in the discussion about the nature of these tetrameric complexes and their potential additional members (e.g., FUL/AP1 or SEP1-3 clades). Ideally, supporting these hypotheses with existing genomic data would further strengthen the work.

Beyond SEP4 genes, this study also addresses a critical component of the GRN governing inflorescence development. The role of AN is especially intriguing, as the an single mutant phenocopies the j2 ej2 lin triple mutant. The data show that AN expression is regulated by the SEPs (important new result), but it remains unclear how much of the triple sep4 mutant phenotype can be attributed to this regulation.

The authors propose that FA acts upstream of SEP4 initially and, later—once AN is induced by SEP4 proteins—FA collaborates with AN and acts upstream of SEP1-3. A positive feedback loop between FA/AN and SEP4 is suggested to maintain the floral state. This section is highly compelling, as it bridges various classes of MADS-box transcription factors with FA/AN, addressing a notable gap in the field. It also contrasts with the situation in petunia, another Solanaceae species that shares many features but exhibits key differences.

What I find most lacking in this work is an analysis using in situ hybridization to spatially validate the main inferred regulatory interactions: the three SEP4 genes in wild-type vs. fa backgrounds, FA and AN in the triple sep4 mutant, and fa and the three sep4 genes in the an background. Some of these data may already exist in the literature and could be incorporated.

Finally, a critical question remains: Why are both SEP4 and FA/AN required to initiate flower meristem development and SEP1-3 induction? In situ hybridization experiments (e.g., SEP1-3 in fa, an, and triple sep4 backgrounds) could be performed or at least discussed. Given what is known in other plants—and the observation (line 386) that no J2 binding sites are found in SEP1-3—I would not be surprised if FA/AN or FA dimers serve as the primary upstream regulators.

Minor comments:

* A summary schematic (GRN) highlighting the newly identified regulatory interactions—and the confidence level in each—would be a valuable addition to the final figure.

* The excellent reviews by Claire Perilleux's team could be cited in the introduction and discussion.

* The recent work by Jiang et al. (2025, New Phytologist) should also be cited, particularly as the MADS-box binding in FA might involve regulation by AP1/FUL genes.

Reviewer #3: This manuscript presents some beautiful mutant phenotypes of the three SEP4 paralogs in tomato (including single mutants and various combinatorial mutants) as well as paralogs of the three SEP clades (TM5/TM29/SEP4). Additional transcriptomic and functional data suggest that the SEP proteins directly regulate AN/UFO and FA/LEAFY expression. The latter point is significant because UFO regulation is still poorly understood, despite its critical importance in inflorescence/flower development.

Although I find the data valuable and the figures appealing, the conceptual framework of the paper seems odd: isn't it already well established that genetic synergy usually arises from functional redundancy? This study doesn't really contribute anything new in that regard. I would encourage the authors to reframe the study, perhaps just simply "dissecting the genetic redundancy of the SEP genes and their target genes".

A couple of minor comments:

line 27-29: Again, functional redundancy is the most straightforward explanation of genetic synergy. This sentence is trying hard to package the results of this study in a way that sounds different, but in essence it's still the same as "functional redundancy".

line 359-362: The strength of the synergistic effects probably depends more on the degree of expression overlap between the paralogs instead of evolutionary distance.

# Author comment: Author response to Decision Letter 2

**DOI:** 10.1371/journal.pbio.3003946.r003

27 May 2026

Attachment Submitted filename: Gaarslev-etal-ReviewerResponse_R1.pdf

# Peer review: Decision Letter 2

**DOI:** 10.1371/journal.pbio.3003946.r004

**Authors:** Ankiit Ahluwalia

10 Jul 2026

Dear Dr Soyk,

Thank you for your patience while we considered your revised manuscript "Incomplete functional divergence drives MADS-box gene synergy during floral development in tomato" for publication as a Research Article at PLOS Biology. This revised version of your manuscript has been evaluated by the PLOS Biology editors, the Academic Editor and the original reviewers.

Based on the reviews, we are likely to accept this manuscript for publication, provided you satisfactorily address the remaining points (if any) raised by the reviewers. Please also make sure to address the following data and other policy-related requests.

IMPORTANT - please attend to the following:

a) Please address my Data Policy requests below; specifically, we need you to supply the numerical values underlying Fig. 2C, H, I, J; Fig. 3A-D, G, H; Fig. 4A, B, D, E, G, I, J; Fig. 5A; Fig. 6J, L, M, N; Fig. S1A; Fig. S2A, B, C, D; Fig. S4A, L; Fig. S5C; Fig. S6A-C; Fig. S7A, B either as a supplementary data file or as a permanent DOI’d deposition (such as Zenodo).

b) Please clearly cite the location of the data in all relevant main and supplementary figure legends, for example: “The data underlying this figure can be found at doi.org/10.xxx/zenodo.xxxxx.” We understand that this may appear repetitive, but it helps make the figures and their legends more self contained.

c) Please make the raw data deposited on SRA publicly available at this stage.

d) Please make any custom code available (if any), either as a supplementary file or as part of your data deposition.

As you address these items, please take this last chance to review your reference list to ensure that it is complete and correct. If you have cited papers that have been retracted, please include the rationale for doing so in the manuscript text, or remove these references and replace them with relevant current references. Any changes to the reference list should be mentioned in the cover letter that accompanies your revised manuscript.

In addition to these revisions, you may need to complete some formatting changes, which you will receive in a follow up email. A member of our team will be in touch with a set of requests shortly. If you do not receive a separate email within a few days, please assume that checks have been completed, and no additional changes are required.

We expect to receive your revised manuscript within two weeks.

To submit your revision, please go to https://www.editorialmanager.com/pbiology/ and log in as an Author. Click the link labelled 'Submissions Needing Revision' to find your submission record. Your revised submission must include the following:

- a cover letter that should detail your responses to any editorial requests, if applicable, and whether changes have been made to the reference list

- a Response to Reviewers file that provides a detailed response to the reviewers' comments (if applicable, if not applicable please do not delete your existing 'Response to Reviewers' file.)

- a track-changes file indicating any changes that you have made to the manuscript.

NOTE: If Supporting Information files are included with your article, note that these are not copyedited and will be published as they are submitted. Please ensure that these files are legible and of high quality (at least 300 dpi) in an easily accessible file format. For this reason, please be aware that any references listed in an SI file will not be indexed. For more information, see our Supporting Information guidelines:

https://journals.plos.org/plosbiology/s/supporting-information

*Published Peer Review History*

Please note that you may have the opportunity to make the peer review history publicly available. The record will include editor decision letters (with reviews) and your responses to reviewer comments. If eligible, we will contact you to opt in or out. Please see here for more details:

https://plos.org/published-peer-review-history/

*Press*

Should you, your institution's press office or the journal office choose to press release your paper, please ensure you have opted out of Early Article Posting on the submission form. We ask that you notify us as soon as possible if you or your institution is planning to press release the article.

*Protocols deposition*

To enhance the reproducibility of your results, we recommend that if applicable you deposit your laboratory protocols in protocols.io, where a protocol can be assigned its own identifier (DOI) such that it can be cited independently in the future. Additionally, PLOS ONE offers an option for publishing peer-reviewed Lab Protocol articles, which describe protocols hosted on protocols.io. Read more information on sharing protocols at https://plos.org/protocols?utm_medium=editorial-email&utm_source=authorletters&utm_campaign=protocols

Please do not hesitate to contact me should you have any questions.

Sincerely,

Ankiit

Ankiit Ahluwalia,

aahluwalia@plos.org

PLOS Biology

------------------------------------------------------------------------

****[DELETE AS APPROPRIATE]****

ETHICS STATEMENT:

-- Please include the full name of the IACUC/ethics committee that reviewed and approved the animal care and use protocol/permit/project license. Please also include an approval number.

-- Please include the specific national or international regulations/guidelines to which your animal care and use protocol adhered. Please note that institutional or accreditation organization guidelines (such as AAALAC) do not meet this requirement.

-- Please include information about the form of consent (written/oral) given for research involving human participants. All research involving human participants must have been approved by the authors' Institutional Review Board (IRB) or an equivalent committee, and must have been conducted according to the principles expressed in the Declaration of Helsinki.

------------------------------------------------------------------------

DATA POLICY:

You may be aware of the PLOS Data Policy, which requires that all data be made available without restriction: http://journals.plos.org/plosbiology/s/data-availability. For more information, please also see this editorial: http://dx.doi.org/10.1371/journal.pbio.1001797

Note that we do not require all raw data. Rather, we ask that all individual quantitative observations that underlie the data summarized in the figures and results of your paper be made available in one of the following forms:

1) Supplementary files (e.g., excel). Please ensure that all data files are uploaded as 'Supporting Information' and are invariably referred to (in the manuscript, figure legends, and the Description field when uploading your files) using the following format verbatim: S1 Data, S2 Data, etc. Multiple panels of a single or even several figures can be included as multiple sheets in one excel file that is saved using exactly the following convention: S1_Data.xlsx (using an underscore).

2) Deposition in a publicly available repository. Please also provide the accession code or a reviewer link so that we may view your data before publication.

Regardless of the method selected, please ensure that you provide the individual numerical values that underlie the summary data displayed in the following figure panels as they are essential for readers to assess your analysis and to reproduce it:

[Figs….]

NOTE: the numerical data provided should include all replicates AND the way in which the plotted mean and errors were derived (it should not present only the mean/average values).

Please also ensure that figure legends in your manuscript include information on where the underlying data can be found, and ensure your supplemental data file/s has a legend.

Please ensure that your Data Statement in the submission system accurately describes where your data can be found.

------------------------------------------------------------------------

APPROPRIATE DATA PRESENTATION?

(If you think the paper is not using best practice, add the following)

- Please ensure that you are using best practice for statistical reporting and data presentation. These are our guidelines https://journals.plos.org/plosbiology/s/best-practices-in-research-reporting#loc-statistical-reporting and a useful resource on data presentation https://journals.plos.org/plosbiology/article?id=10.1371/journal.pbio.1002128

- If you are reporting experiments where n ≤ 5, please plot each individual data point.

------------------------------------------------------------------------

CODE POLICY

Per journal policy, if you have generated any custom code during the course of this investigation, please make it available without restrictions. Please ensure that the code is sufficiently well documented and reusable, and that your Data Statement in the Editorial Manager submission system accurately describes where your code can be found. More information on our Code Policy, what and how to share can be found here: https://journals.plos.org/plosbiology/s/code-availability

Please note that we cannot accept sole deposition of code in GitHub, as this could be changed after publication. However, you can archive this version of your publicly available GitHub code to Zenodo. Once you do this, it will generate a DOI number, which you will need to provide in the Data Accessibility Statement (you are welcome to also provide the GitHub access information). See the process for doing this here: https://docs.github.com/en/repositories/archiving-a-github-repository/referencing-and-citing-content

------------------------------------------------------------------------

SPECIES INDICATED IN THE ABSTRACT?

- Please note that per journal policy, the model system/species studied should be clearly stated in the abstract of your manuscript.

**EDITOR, CONSIDER ALSO INCLUDING ORGANISM IN THE TITLE, IF SENSICAL/RELEVANT AND/OR TO AVOID BEING MISLEADING/OVERREACHING (not always preferred)**

------------------------------------------------------------------------

BLOT AND GEL REPORTING REQUIREMENTS:

We require the original, uncropped and minimally adjusted images supporting all blot and gel results reported in an article's figures or Supporting Information files. We will require these files before a manuscript can be accepted so please prepare and upload them now. Please carefully read our guidelines for how to prepare and upload this data: https://journals.plos.org/plosbiology/s/figures#loc-blot-and-gel-reporting-requirements

------------------------------------------------------------------------

DATA NOT SHOWN?

- Please note that per journal policy, we do not allow the mention of "data not shown", "personal communication", "manuscript in preparation" or other references to data that is not publicly available or contained within this manuscript. Please either remove mention of these data or provide figures presenting the results and the data underlying the figure(s).

------------------------------------------------------------------------

Reviewer remarks:

Reviewer #1: The authors have done a thorough job addressing each of the reviewers' comments, both through the additional of new data/figures/text or arguing their case. I'm happy with the changes and believe this paper will make a strong contribution to our understanding of functional divergence, MADS-box gene evolution, and mechanisms of plant development.

Reviewer #2: I have read with interest the new version of this manuscript as well as the responses to the referees. I already had a very good opinion of the first version, and I asked for a few things without claiming any of them would be absolutely required. I am very satisfied with the new version. I appreciate that the title was slightly amended. I think the addition of the RNA-seq in sep4t and an add a lot in terms of regulatory network and largely compensate for the lack of in situ hybridization. Moreover, I think the little summary scheme presented in Figure 6 is very useful for the readers. Overall, I think this work represents a great contribution to the field of inflorescence and flower development in tomato and in angiosperms in general.Little detail: I had in mind the tomato review by Perilleux et al. (https://doi.org/10.3389/fpls.2022.798502), even if the Arabidopsis reviews are also of high quality.

Reviewer #3: The authors did a great job in revising the manuscript and addressed my comments satisfactorily.

# Author comment: Author response to Decision Letter 3

**DOI:** 10.1371/journal.pbio.3003946.r005

17 Jul 2026

Attachment Submitted filename: Gaarslev-etal-ReviewerResponse_R2.docx

# Editor report: Decision Letter 3

**DOI:** 10.1371/journal.pbio.3003946.r006

**Authors:** Ankiit Ahluwalia

28 Jul 2026

Dear Dr Soyk,

Thank you for the submission of your revised Research Article "Incomplete functional divergence drives MADS-box gene synergy during floral development in tomato" for publication in PLOS Biology. On behalf of my colleagues and the Academic Editor, Alexander Jones, I am pleased to say that we can in principle accept your manuscript for publication, provided you address any remaining formatting and reporting issues. These will be detailed in an email you should receive within 2-3 business days from our colleagues in the journal operations team; no action is required from you until then. Please note that we will not be able to formally accept your manuscript and schedule it for publication until you have completed any requested changes.

Please take a minute to log into Editorial Manager at http://www.editorialmanager.com/pbiology/, click the "Update My Information" link at the top of the page, and update your user information to ensure an efficient production process.

PRESS

We frequently collaborate with press offices. If your institution or institutions have a press office, please notify them about your upcoming paper at this point, to enable them to help maximise its impact. If the press office is planning to promote your findings, we would be grateful if they could coordinate with biologypress@plos.org. If you have previously opted in to the early version process, we ask that you notify us immediately of any press plans so that we may opt out on your behalf.

We also ask that you take this opportunity to read our Embargo Policy regarding the discussion, promotion and media coverage of work that is yet to be published by PLOS. As your manuscript is not yet published, it is bound by the conditions of our Embargo Policy. Please be aware that this policy is in place both to ensure that any press coverage of your article is fully substantiated and to provide a direct link between such coverage and the published work. For full details of our Embargo Policy, please visit http://www.plos.org/about/media-inquiries/embargo-policy/.

Thank you again for choosing PLOS Biology for publication and supporting Open Access publishing. We look forward to publishing your study.

Sincerely,

Ankiit Ahluwalia,

PLOS Biology

aahluwalia@plos.org
